# Australian strains of the avian coronavirus infectious bronchitis virus predominantly target the respiratory tract rather than the kidneys in specific-pathogen-free chickens

**DOI:** 10.1099/jgv.0.002213

**Published:** 2026-01-23

**Authors:** Jose A. Quinteros, Panayiotis Loukopoulos, Amir H. Noormohammadi, Glenn F. Browning, Mauricio J. C. Coppo, Paola K. Vaz, Alistair R. Legione, Omid Fahri, Dulari S. Thilakarathne, Adepeju E. Onasanya, Gayathri Gopakumar, Marzieh Armat, Andrés Diaz-Méndez

**Affiliations:** 1 Asia-Pacific Centre for Animal Health, Melbourne Veterinary School, Faculty of Science, The University of Melbourne, Parkville, 3010, Victoria, Australia; 2 Poultry Research Foundation, Sydney School of Veterinary Science, Faculty of Science, The University of Sydney, Camden, 2570, New South Wales, Australia; 3 Asia-Pacific Centre for Animal Health, Melbourne Veterinary School, Faculty of Science, The University of Melbourne, Werribee, 3030, Victoria, Australia; 4 Instituto One Health, Facultad de Ciencias de la Vida, Universidad Andres bello, Concepcion, Biobio, Chile; 5 Department of Veterinary Pathobiology, Faculty of Veterinary Medicine & Animal Science, University of Peradeniya, Peradeniya, Sri Lanka

**Keywords:** attenuation, avian coronavirus, infectious bronchitis virus (IBV), pathogenicity, virulence

## Abstract

Coronaviruses evolve rapidly, with recombination and mutation fostering the emergence of variant strains. The avian coronavirus infectious bronchitis virus (IBV) is an important poultry pathogen and a valuable natural model for studying coronaviruses. Australian strains have evolved independently of those infecting chickens elsewhere in the world, so understanding the biology and evolution of these strains can further our understanding of factors driving the emergence of novel coronaviruses. We infected groups of specific pathogen-free Leghorn chickens with six Australian IBVs (from five distinct genotypes) isolated between 1962 and 2013. All six affected the respiratory tract, but only one was nephropathogenic (N1/62). All six induced significant lesions and actively replicated in the upper respiratory tract, but they had lower levels of replication and induced less severe lesions in the middle and lower trachea. There were significant differences between the six strains in the severity of the lesions they induced and in their tissue tropism and effect on tracheal ciliary motility. Strains N1/62 (strain T) and N1/03 caused the most severe tracheal ciliostasis and replicated to the highest levels in tissues. Strain N1/03 caused the most severe lesions at 9 days post-infection. Only strain N1/03 caused lesions in the lower trachea. Overall, strains N1/03 and N1/62 were the most virulent. This study is the first to characterize the histological changes induced by the recently isolated Australian IBVs and compare them directly with older strains. Recombination between field and vaccine strains of IBV has yielded emergent IBVs in Australia that appear to have enhanced virulence for the respiratory tract.

## Introduction

One of the lessons learned during the Severe Acute Respiratory Syndrome Coronavirus 2 SARS-CoV-2 pandemic is that the rapid evolution of coronaviruses and the constant emergence of new variants can considerably impact disease control [1,3]. However, this observation is far from novel. Over the last 90 years, many variant strains of the avian coronavirus infectious bronchitis virus (IBV) have been isolated from chickens in different parts of the world [4,6].

Infection with IBV can cause histopathological changes in the trachea and the kidneys. The changes in the trachea may include ciliary loss, oedema, haemorrhage, epithelial and mucous gland degeneration, hyperplasia, lymphocytic infiltration and aggregation and hypertrophy of the alveolar mucous glands [7,9]. The changes in the kidneys may include lymphocytic infiltration, degeneration of the tubular epithelium, ductal and tubular dilation, renal haemorrhage and oedema of Bowman’s capsule [7,9, 10].

Because of geographical isolation and barriers to live chicken imports for decades, IBVs in Australia have had an evolutionary path that appears distinct from those seen elsewhere [11,14]. This facilitates studies of their evolutionary trends and the molecular mechanisms underlying them.

Australian strains of IBV have been grouped into six different genotypes: GI-5, GI-6, GIII-1, GIII-2, GIII-3 and GV-1 [12,15]. They have also been historically named following the nomenclature proposed by Cavanagh [16] and Jackwood [6], SI/YY, where S refers to the state where it was isolated, I the isolate number and YY the year of isolation. Under that nomenclature, the name of the strain N1/08 indicates that it was the first isolate from New South Wales samples in 2008 [14].

Even though the pathogenicity of some strains has been assessed previously, in most cases, these analyses have been incomplete. In 2002, 25 Australian isolates of IBV dating from 1962 to 1994 were characterized [17]. Groups of 2-week-old birds were infected with 10^4^ median ciliostatic doses (CD_50_) of each strain. Then, clinical scores, viral replication and histopathological changes in the trachea and the kidneys were assessed. In 2014, the pathogenicity of strain N1/08 was evaluated [18]. In Hewson *et al*. [18], 2-week-old specific pathogen-free (SPF) chickens were infected with 3.2×10^5^ median embryo infectious doses (EID_50_) of the virus. Clinical signs were evaluated and scored daily in the chickens in one group, while the birds in a second group were sequentially euthanized. Viral replication and histopathological changes in the trachea, kidneys and caecal tonsils were assessed. While histopathological lesions in the trachea and kidney had been assessed for some of the older strains in these previous studies mentioned above, this aspect has not been evaluated for the more recent strains (i.e. those that have been isolated after 2002).

The studies described in the present manuscript aimed to compare the pathogenicity and tissue tropism of historical and contemporary Australian isolates of IBV in SPF chickens. The complete genome sequences of these viruses were used to select the strains to ensure that the full diversity of Australian genotypes of IBV was included. The proportions of chickens that had tracheal ciliostasis were determined and compared to assess upper respiratory tract pathogenicity. Therefore, a complete comparative assessment was performed of the histopathological lesions induced by older strains and the previously uncharacterized isolates. Particularly, Q1/13, the most recent isolate at the time of the design of this study, was isolated from a 2013 outbreak in Queensland, Australia.

## Methods

### Propagation and purification of viral strains

The archived strains used in this study, N1/62 (also known as strain T or T-strain), Q1/73, V5/90, N1/03 and N1/08, were isolated between 1962 and 2008, propagated in SPF embryonated hen eggs (Australian SPF Services) and stored as allantoic fluid (AF) at −80 °C. For propagation, the frozen allantoic fluid was thawed and 100 µl was inoculated into the allantoic cavity of 9-day-old SPF embryonated hen eggs. At 48 h after inoculation, the eggs were placed at 4 °C for at least 6 h to kill the embryos, and the AF was collected aseptically in a Class II Biosafety Cabinet. The AFs were aliquoted into sterile 2 ml screw-cap tubes (500 µl each) and stored at −80 °C.

A further strain was isolated from the caecal tonsils of a broiler chicken from a farm located in Queensland during an IBV outbreak in 2013 and was designated Q1/13. The tissue was homogenized using a sterile mortar, pestle and sand in viral transport medium (VTM) consisting of Dulbecco’s Modified Eagle’s Medium (Thermo Fisher Scientific, Australia) supplemented with 1% FBS (Sigma-Aldrich, Australia) and 50 µg ml^−1^ gentamicin. This homogenate was then clarified by centrifugation at 2,500 ***g*** for 20 min at 4 °C. The supernatant was then collected and filtered through a 0.45-µm syringe filter (Millex-HV, Merck Millipore). The filtrate (500 µl) was inoculated into the allantoic cavity of four 9-day-old SPF embryonated eggs. After 48 h, the AF was collected aseptically and tested using a conventional reverse transcriptase-PCR assay targeting the 3′ UTR and the nucleocapsid protein gene (N-3′ UTR) of the virus [19]. The positive IBV AFs were subjected to four blind serial passages in the allantoic cavity of SPF eggs, as described above. Each passage was aliquoted into sterile 2-ml screw-cap tubes (500 µl in each) and stored at −80 °C.

For genomic sequencing and preparation of inocula for experimental infection studies, each strain was propagated once in the allantoic cavities of five embryonated SPF eggs. AFs were collected aseptically, pooled and homogenized, yielding a total volume of ~40 ml per strain. The pooled AFs for each strain were tested for bacterial contamination by inoculation onto sheep blood agar plates that were incubated at 37 °C for 60 h. The AFs were clarified by centrifugation at 2,500 ***g*** for 20 min at 4 °C, and the supernatant was then centrifuged at 100,000 ***g*** for 2 h at 4 °C to pellet the virions. The pellet was resuspended in 200 µl of magnesium- and calcium-ion-free PBS, pH 7.4. This is also known as Dulbecco’s PBS [20]. The viral suspension was then overlaid on a 30–55% continuous sucrose gradient and centrifuged at 100,000 ***g*** for 4 h at 4 °C. The visible viral band formed in the gradient was harvested directly from the tube using an 18-gauge needle, transferred to sterile 1.5-ml microcentrifuge tubes and stored at 4 °C. The harvests from the gradient were mixed and resuspended in Dulbecco’s PBS and centrifuged at 90,000 ***g*** for 1 h at 4 °C. The viral pellet was resuspended in 250 µl of PBS-A and stored at −80 °C until further use.

A recombination analysis was conducted using the complete genome sequence of the six strains from this *in vivo* study and three available Australian vaccine strains. The analysis was performed in RDP4 version 4.101 [21], with default settings and a multiple alignment generated using the Clustal Omega Plugin available in the Geneious Prime^®^ 2025.2.2 (https://www.geneious.com) software package. Nine detection methods were applied: RDP, GENECONV, Bootscan, Maxchi, Chimaera, SiScan, PhylPro, Lard and 3Seq. A recombinant was considered significant only if detected by five or more methods. Recombination events involving a vaccine strain as the recombinant were excluded.

### Sequencing of the viral genomes

The genomes of 13 Australian strains of IBV were sequenced to identify representatives of all the Australian genotypes of IBV. Genomic RNA was extracted from the purified IBV strains using RNeasy mini kits (QIAGEN), following the manufacturer’s instructions for cell culture and tissue samples, with slight modifications, as described in Quinteros *et al*. [13]. The extracted RNA was treated with Turbo DNase (Thermo Fisher Scientific), following the manufacturer’s instructions. RNA samples from all the strains, except for V18/91 and V1/07, were submitted to the Hudson Institute (Monash University, Australia) for next-generation sequencing using the Illumina MiSeq system, generating 300 bp paired-end reads. The viral RNA libraries were prepared using the Illumina TruSeq Stranded mRNA kit. For strains V18/91 and V1/07, sequencing was performed as described in Quinteros *et al*. [13], using the PGM Ion Torrent platform (Life Technologies), with a 314 chip and the 200-base sequencing Ion OneTouch Kit v2. All sequences were determined using the trimmed and normalized reads, which were assembled using the SPAdes *de novo* assembler [22].

### Experimental infection of SPF chickens

**Fig. 1.**
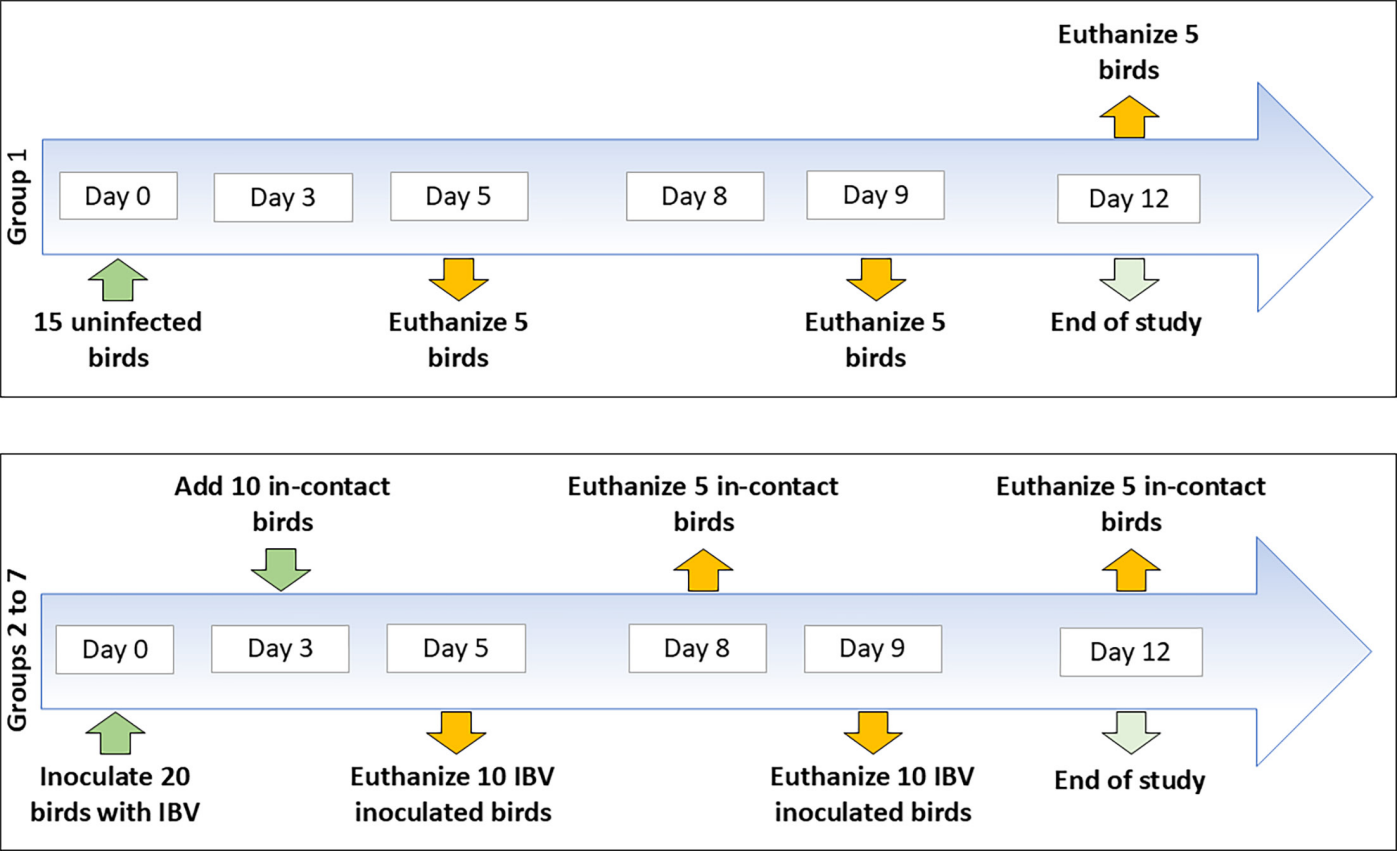
Outline of the experimental infection study, showing the allocation of birds to each group, including control, infected and in-contact birds. The days of infection and euthanasia are indicated with arrows.

The Animal Ethics Committee of the University of Melbourne approved the use of chickens in this experiment under approval 181450.1. The experimental infection studies are outlined in Fig. 1. A total of 195 2-week-old SPF chickens were randomly assigned to seven groups. Fifteen chickens were allocated to group 1 (uninfected control group), and 20 birds were initially assigned to each of groups 2 to 7. Each group was housed in an isolator equipped with a HEPA filter and provided with water and feed *ad libitum*. Chickens in group 1 were inoculated with VTM prepared as described above. Chickens in groups 2 to 7 (infected groups) were inoculated with 1.4×10^4^ EID_50_ of the strains Q1/73, genotype GI-6 (group 2); Q1/13, genotype GV (group 3); N1/62, genotype GI-5 (group 4); N1/03, genotype GV (group 5); V5/90, genotype GI-6 (group 6); and N1/08, genotype GIII-1 (group 7). Of the genotypes that have been detected in Australian chicken flocks, only GIII-2 and GIII-3 were not represented. On day 3 post-inoculation (3 DPI), 10 additional naïve SPF birds were introduced into each of groups 2 to 7 to serve as in-contact birds.

At each euthanasia endpoint [5 and 9 DPI or days post-contact (DPC) for the in-contact chickens], birds were euthanized by exposure to an overdose of halothane. Cross sections of the upper trachea (tracheal rings) were aseptically collected into 3 ml of VTM and immediately transferred to a 5% (v/v) CO2 in air incubator at 37 °C to assess ciliostasis. Sections of the upper, middle (mid) and lower trachea, and the kidney, were fixed in formalin, embedded in wax (Paraplast, Sigma-Aldrich) and processed to produce 3-µm-thick haematoxylin and eosin-stained sections. Histopathological examinations and scoring were performed by a veterinary pathologist blinded to the treatments each bird had received, and the slides were provided for examination in random order. Swabs of the conjunctiva, sections of the trachea and proventriculus and the caecal tonsils and left kidney were collected and placed in 2-ml tubes containing VTM on ice and posteriorly transferred to −80 °C until further processing.

### Histopathological evaluation of the trachea

The parameters assessed were lesion chronicity and severity (Table 1) and tracheal mucosal thickness.

**Table 1.** The grading system used for the classification of the chronicity and severity of tracheal lesions in IBV-infected chickens

Stage	Histopathological change	Grade of lesion
Acute	Sloughing/oedema/capillary congestion/deciliation	0=absent 1=mild 2=moderate 3=severe 4=very severe
Subacute	Epithelial hyperplasia/metaplasia	
Chronic	Lymphocytic infiltration/lymphoid follicle formation	

All grades from 0 to 4 apply for each stage of disease (acute, subacute and chronic).

**Fig. 2.**
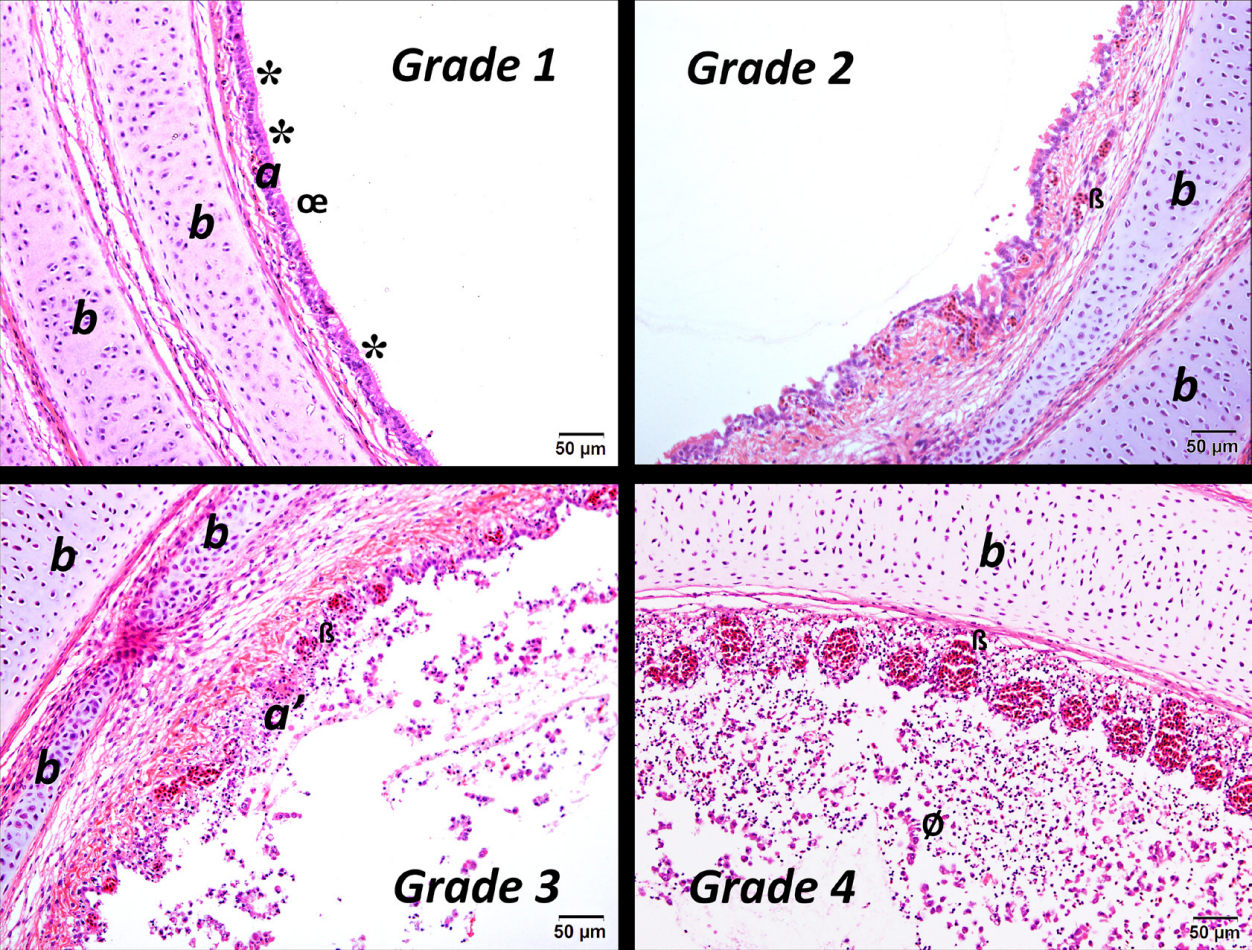
Mid-tracheal sections of birds infected with IBV with acute lesions of varying severity (grades 1 to 4); a, epithelium; a′, sloughing/oedema of the epithelium; b, tracheal cartilage. *, areas of the epithelium where the cilia have been lost; œ, sloughing of the cilia; ß, congestion of the capillaries of the lamina propria; ø, portion of epithelium detached from the trachea. Stained with haematoxylin and eosin. Bar=50 µm.

Lesions were classified as follows: acute (A) if there was evidence of deciliation, oedema, rounding and sloughing of the epithelial cells and minor infiltration of heterophils; subacute (SA) if there was hyperplasia and/or metaplasia of the epithelia; and as chronic (C) if there was infiltration of the lamina propria with lymphocytes and/or formation of germinal centres [23,24]. The severity of the lesions was also graded as absent (0), mild (1), moderate (2), severe (3) or very severe (4). Images of lesions representative of the different grades are shown in Figs2^4^.

**Fig. 3.**
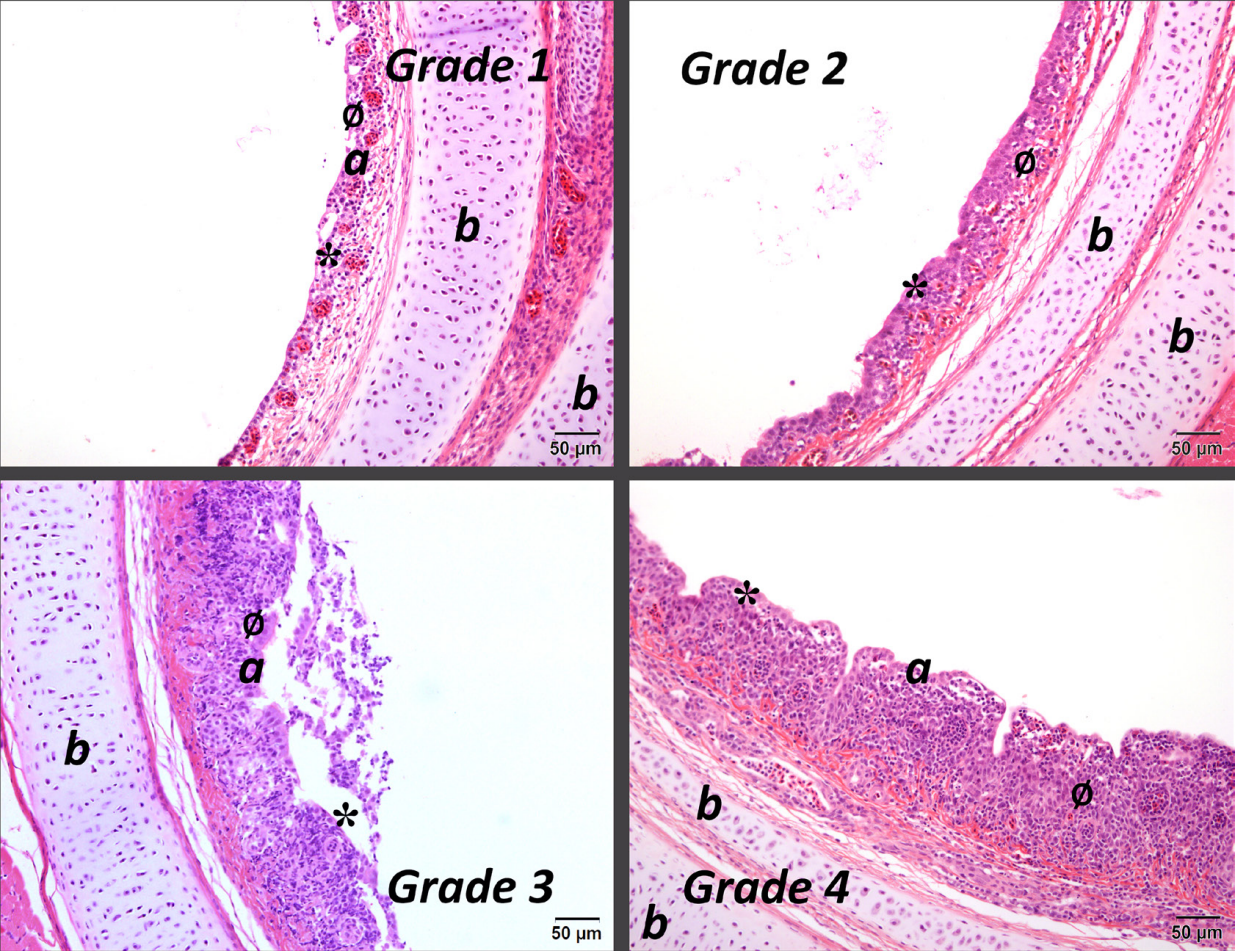
Mid-tracheal sections of birds infected with IBV with subacute lesions of varying severity (grades 1 to 4); a, epithelium; b, tracheal cartilage. * indicates areas of metaplastic epithelium; ø indicates areas of hyperplastic epithelium. Stained with haematoxylin and eosin. Bar=50 µm.

**Fig. 4.**
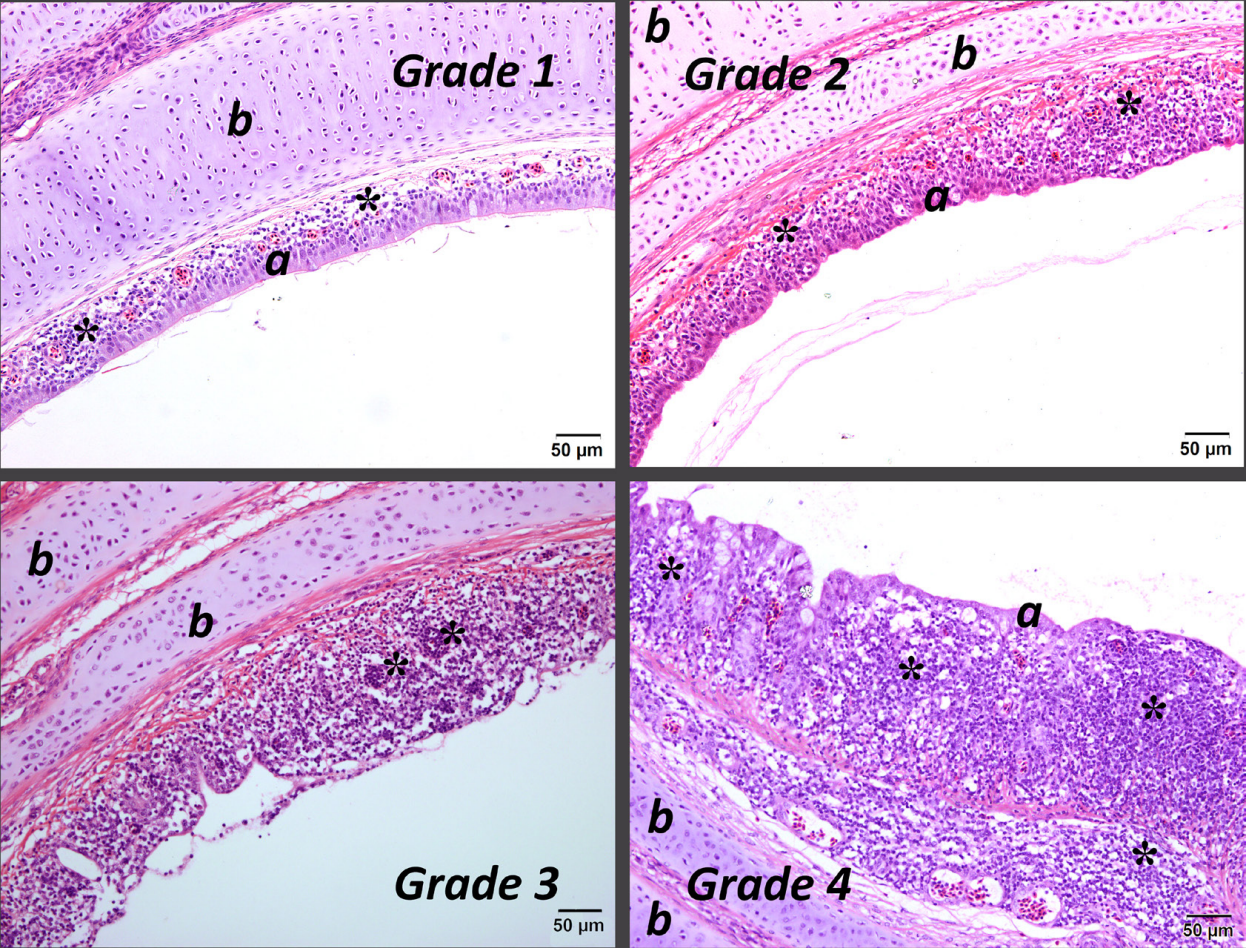
Mid-tracheal sections of birds infected with IBV with chronic lesions of varying severity (grades 1 to 4) a, epithelium; b, tracheal cartilage. *, areas of lymphocytic infiltration. Stained with haematoxylin and eosin. Bar=50 µm.

The thickness of the mucosae of the three tracheal sections from each bird was also measured. Each tracheal section (upper, mid and lower trachea) was measured at four random cross-sectional points, and the average of the four measurements for each section was calculated. The thickness of the tracheal mucosa was compared between groups using the data collected from both infected and in-contact chickens at 5 and 9 DPI and DPC.

### Histopathological evaluation of the kidneys

**Fig. 5.**
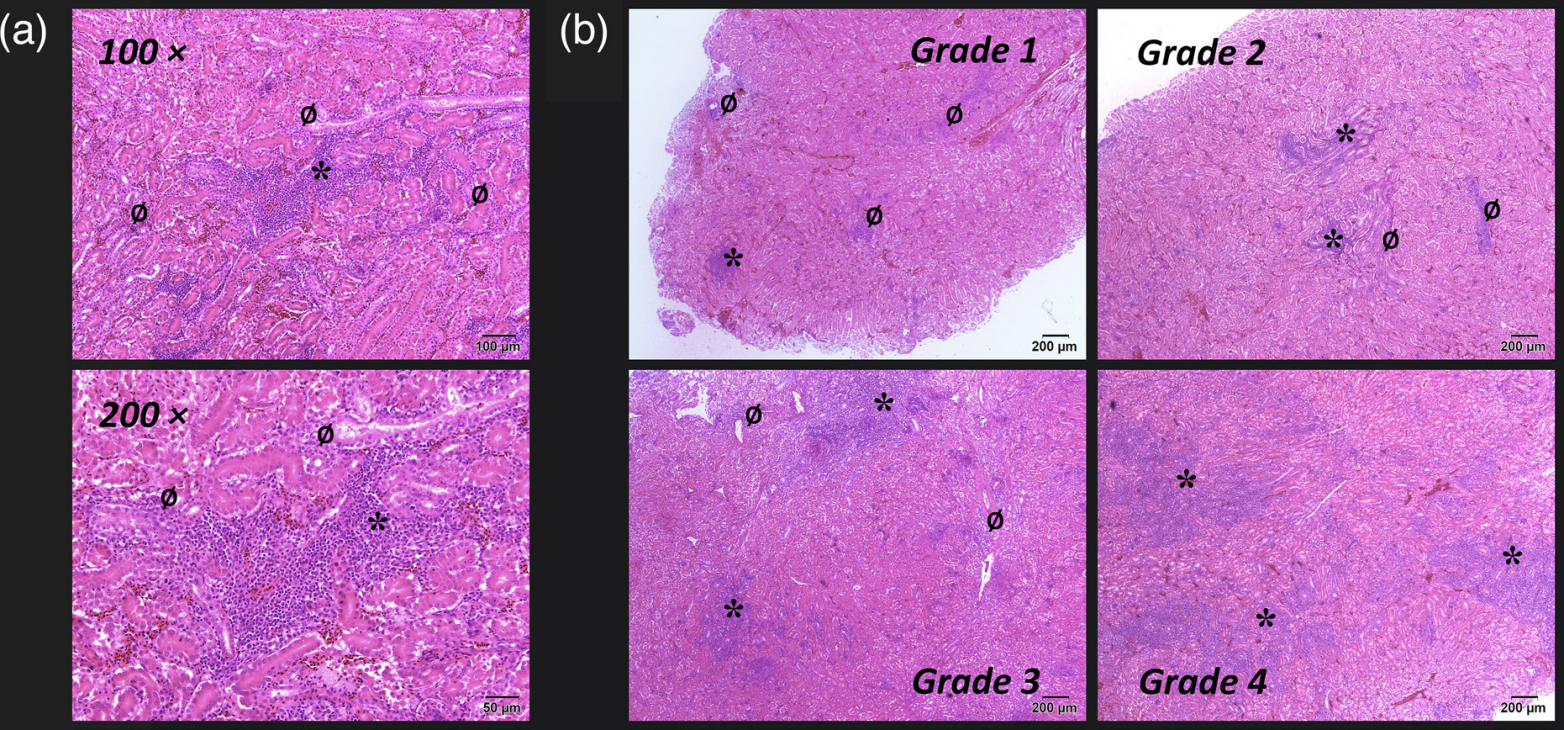
Renal changes in IBV-infected chickens. (**a**) Uninfected; the same kidney section at 100× and 200× magnification showing embryonic rests, a normal histological feature, to highlight the differences between embryonic rests and the areas of lymphocytic infiltration; (**b**) examples of tissue sections with increasingly severe histological changes, corresponding to different histopathological grades for renal changes. *, foci of lymphocytic infiltration; ø, embryonic rests. Magnification, middle and right panels – 50 ×; left panels, as indicated within the panel. Stained with haematoxylin and eosin.

For the evaluation of the kidneys, the lesions were classified based on the degree of lymphocytic infiltration of the renal tissue (Table 2 and Fig. 5). Images representative of the different degrees of lymphocytic infiltration and corresponding grades are provided in Fig. 5b. Other changes associated with infection with IBV, including tubular degeneration, urate accumulation and heterophilic infiltration [23], were not observed in the present study and, hence, were not assessed.

**Table 2.** Criteria for histopathological grading of renal lesions in chickens infected with IBV

Histopathological change	Grade
No lymphocytic infiltration	0
One or two small foci of lymphocytic infiltration	1
Several small foci/one or two extensive areas of lymphocytic infiltration	2
Multiple areas of extensive lymphocytic infiltration	3
Diffuse severe lymphocytic infiltration	4

### Evaluation of tracheal ciliostasis

Tracheal sections in VTM were examined at 40× magnification using an inverted microscope. The presence or absence of ciliostasis was evaluated and recorded. If ciliary movement was observed, the sample was classified as negative for ciliostasis (video 1, available in the online Supplementary Material). If ciliary movement was not observed (100% of cilia were static), the sample was classified as positive for ciliostasis (video 2, available in the online Supplementary Material

). Due to the large number of tracheal samples collected for other analysis [viral genome quantification (swabs) and histopathology (sections fixed in formalin)], only a single tracheal section was available for evaluating ciliostasis.

### Viral genome copy number quantitation

Total RNA was extracted from tissue samples (conjunctiva, different sections of the trachea, kidney, proventriculus and caecal tonsil swabs) using the MagMAX CORE Nucleic Acid Purification Kit (Thermo Fisher Scientific) and the KingFisher Flex Purification System (Thermo Fisher Scientific) using 50 µl of elution buffer. After extraction, the elution plates were covered with PureLink Foil Tape (Thermo Fisher Scientific) and stored at −80 °C until further use.

RNA extracted from each of the tissue samples was subjected to reverse transcriptase quantitative PCR (RTqPCR) to quantify the viral load, and the chicken glyceraldehyde 3-phosphate dehydrogenase (chGAPDH) gene will be used as a reference. For all the reverse transcription and the qPCRs, the reaction plates were prepared using a QIAgility automated PCR setup machine (QIAGEN). The plates containing the RNA extracts were thawed on ice and centrifuged in an Allegra X-12R centrifuge (Beckman Coulter) at 500 ***g*** for 1 min. The cDNA was generated using SuperScript™ III reverse transcriptase (Thermo Fisher Scientific), following the manufacturer’s instructions. The first reaction mixture was prepared by combining 0.5 mM of dNTPs, 50 nM of oligo(dT)_20_ (Thermo Fisher Scientific), 12.5 µl of the RNA eluate and nuclease-free water to yield a 20 µl reaction volume. The mixture was incubated in a T100 Thermal Cycler (Bio-Rad) at 65 °C for 5 min to denature the RNA. The reactions were then immediately transferred onto ice for at least 1 min, and then each reaction was mixed with 4 µl of 5×first-strand buffer (Thermo Fisher Scientific), 20 mM of DTT (Thermo Fisher Scientific), 40 U of RNaseOUT RNase inhibitor (Thermo Fisher Scientific) and 200 U of SuperScript III reverse transcriptase (Thermo Fisher Scientific). The reactions were transferred to the thermocycler and incubated at 55 °C for 1 h and then at 70 °C for 15 min. The cDNA was then used immediately for the qPCR assays or stored at −20 °C for further use.

The primers used for this qPCR assay were designed specifically for this study to amplify a region spanning the carboxyl terminal region of the nucleocapsid protein and the first nucleotides of the 3′ UTR (N-3′UTR). The qPCR assays were performed using a Stratagene Mx3000P thermocycler (Agilent). The 20 µl qPCR reaction mixture contained 5 µl of 5×Colourless Flexi Reaction Buffer (Promega), 1.25 µM each of the forward and reverse primers (IVE-F, 5′-CAGGATGATGAAGTAGATAAAGC-3′; IVE-R, 5′-GGTAATCAAGCTACATGCCT-3′), 2.5 mM of MgCl_2_, 250 µM of dNTPs, 0.1 µl of Rox reference dye (Thermo Fisher Scientific), 10 µM of SYTO 9 green fluorescent nucleic acid stain (Thermo Fisher Scientific), 1.25 U of GoTaq Flexi DNA polymerase (Promega) and nuclease-free water to yield the final reaction volume. This reaction mixture was then mixed with 5 µl of cDNA and incubated in a thermocycler. The reactions were initially incubated at 94 °C for 10 min and then through 35 cycles of 94 °C for 20 s, 57 °C for 20 s and 72 °C for 30 s. Finally, the reactions were incubated at 95 °C for 1 min and then at 55 °C for 30 s, and a final ramp at a resolution of 0.5 °C and 5 s of soak time up to 95 °C, then held at 95 °C for 30 s, to generate a dissociation curve for the PCR products in each reaction. The qPCR assays targeting the reference gene chGAPDH were run in a Stratagene Mx3000P thermocycler (Agilent). The 18 µl qPCR reaction mix contained 5 µl of 5×Colourless Flexi Reaction Buffer (Promega), 0.56 µM each of the forward and reverse primers, 2 mM MgCl_2_, 278 µM dNTPs, 0.1 µl of Rox reference dye (Thermo Fisher Scientific), 11.1 µM SYTO 9 green fluorescent nucleic acid stain (Thermo Fisher Scientific), 1.25 U of GoTaq Flexi DNA polymerase (Promega) and nuclease-free water. A 2 µl volume of cDNA was added to this reaction mixture, which was then incubated in a thermocycler. The reactions were incubated at 94 °C for 4 min; then through 40 cycles of 94 °C for 30 s, 55 °C for 30 s and 72 °C for 30 s; and finally at 72 °C for 2 min. The reaction products were then incubated at 95 °C for 1 min and 55 °C for 30 s. The temperature was then ramped at a resolution of 0.5 °C and 5 s of soak time up to 95 °C and then held at 95 °C for 30 s to generate a dissociation curve for the PCR products in each reaction. Tenfold dilutions of a plasmid containing the reference gene amplicon were used to generate a standard curve. To calculate the normalized copy numbers of the viral genome, the arithmetic mean of the copy numbers for two runs of the IBV qPCR assays was calculated for each sample. The number of IBV genome copies per 100,000 copies of the chGAPDH reference gene was then calculated.

### Statistical analysis

The proportions of birds exhibiting ciliostasis were compared using two-sided Fisher’s exact tests. The IBV genome copy numbers in the trachea and kidneys were compared using ANOVAs and Tukey’s multiple comparison tests. The tracheal lesion grades were compared using Kruskal–Wallis and correcting for multiple comparisons using false discovery rate. The normalized IBV genome copy numbers in each tissue sample for the different strains were compared by one-way ANOVA and Tukey’s multiple comparison test. All the analyses were performed using GraphPad Prism version 10.0.0 for Windows (GraphPad Software, Boston, MA, USA, https://www.graphpad.com/), and *P*<0.05 was considered significant.

## Results

### Full genome sequences

The whole-genome sequencing was performed to ensure the correct classification of the strains and to select those to be used in the *in vivo* study. The total length of the nucleotide sequences of the strains used and their accession numbers is presented in Table 3. The genotypes shown in Table 3 are based on the classification used in Quinteros *et al*. [12], in which all the Australian strains were classified into six different genotypes, based on the classification of Valastro *et al*. [15]: GI-5 [reference strain (RS) N1/62], GI-6 (RS VicS), GIII-1 (RS N1/88), GIII-2 (RS Q3/88), GIII-3 (RS V18/91) and GV (RS N1/03). Strains N1/03 and N1/08 are recombinant strains in which the S1 glycoprotein gene sequences are derived from an avian coronavirus of unknown origin [13]. The phylogenetic tree inferred from the complete S1 glycoprotein genome, showing the relative genotyping distances to reference strains as described by Valastro *et al*. [15], is presented in Quinteros *et al*. [12].

**Table 3.** Nucleotide sequence length and accession numbers of the IBV strains sequenced in this study

Strain	Year of isolation	State	Genotype	Sequence length (nt)	GenBank accession number
***N1/62 (Strain T)***	1962	NSW	***GI-5 (RS)***	***27,669***	***MK990808***
V1/71	1971	VIC	GI-5	27,780	MK990811
***Q1/73***	1973	QLD	***GI-6***	***27,661***	***MK990813***
Q3/88	1988	QLD	GIII-2 (RS)	27,145	MK982927
***V5/90***	1990	VIC	***GI-6***	***27,596***	***MK990812***
V18/91	1991	VIC	GIII-3 (RS)	26,938	MK778365
V6/92	1992	VIC	GIII-3	26,974	MK778364
Q4/99	1999	QLD	GI-5	27,901	MK982928
Q1/99	1999	QLD	GI-6	27,594	MK972912
V1/02	2002	VIC	GI-5	27,783	MK990809
N1/03*	2003	NSW	***GV***	***27,626***	***KU556806***
V1/07	2007	VIC	GV	27,648	MK990810
N1/08*	2008	NSW	***GIII-1***	***27,688***	***KU556807***
***Q1/13***	2013	QLD	***GV***	***27,636***	***MK972911***
H104	nd	nd	GI-6	27,661	MK972910

The strains used in the experimental infection study are shown in bold italics. The strains marked with an asterisk were sequenced in previous studies [13].

nd, not determined; NSW, New South Wales; QLD, Queensland; VIC, Victoria.

### Recombination analysis

The results of the recombination analysis are presented in Table 4, arranged from top to bottom according to significance, with the most significant events listed first. All isolates from the twenty-first century (post-2000) were identified as recombinants, with N1/08, Q1/13 and N1/03 among the top eight most significant events. In these recombination events, all vaccine strains appear as either major or minor parental sequences (Table 4, highlighted in red).

**Table 4.** Recombination analysis using the Recombination Detection Program (RDP4) (default settings). Only the results deemed significant with five or more detection methods were included. Results with a vaccine as a recombinant were also excluded. The vaccine strains are highlighted in red font

			Detection method								
Recombinant sequence	Minor parental sequence	Major parental sequence	RDP	GENECONV	Bootscan	Maxchi	Chimaera	SiSscan	PhylPro	LARD	3Seq
N1/08	V5/90	A3 vaccine	2.37E-98	8.29E-221	1.31E-98	2.36E-45	8.97E-31	ns	ns	ns	9.33E-15
	I vaccine	N1/62									
	VicS-v vaccine										
Q1/13	V5/90	Unknown (N1/62)	1.64E-89	1.92E-171	6.92E-108	6.50E-46	ns	3.40E-69	ns	ns	9.33E-15
	Q1/73										
	I vaccine										
	VicS-v vaccine										
N1/03	I vaccine	Unknown (Q1/73)	2.61E-34	7.36E-84	3.21E-15	3.43E-17	1.25E-09	1.15E-56	ns	ns	6.39E-03
^N1/03	VicS-v vaccine	N1/08	1.03E-16	1.80E-58	4.45E-26	3.59E-13	2.40E-12	ns	ns	ns	2.35E-11
	I vaccine	A3 vaccine									
	V5/90										
^N1/08	V5/90	Unknown (Q1/73)	1.95E-20	8.10E-54	1.91E-28	6.29E-11	2.49E-11	ns	ns	ns	9.33E-15
	I vaccine										
	VicS-v vaccine										
^N1/08	N1/03	I vaccine	8.43E-20	6.17E-51	6.57E-15	1.44E-09	5.58E-12	ns	ns	ns	2.61E-03
N1/03	Q1/73	Q1/13	1.94E-18	2.78E-42	6.31E-18	3.73E-08	1.84E-07	ns	ns	ns	9.33E-15
	I vaccine										
	V5/90										
	VicS-v vaccine										
N1/08	V5/90	A3 vaccine	4.55E-16	9.37E-36	9.89E-17	1.77E-09	6.41E-09	ns	ns	ns	1.87E-13
	Q1/73										
	I vaccine										
	VicS-v vaccine										
^Q1/73	N1/62	I vaccine	6.71E-21	ns	1.48E-20	1.43E-13	1.28E-14	1.23E-02	ns	ns	1.87E-14
Q1/13	V5/90	N1/03	1.64E-07	5.30E-20	1.72E-06	2.91E-03	4.50E-04	ns	ns	ns	1.73E-06
	Q1/73										
	I vaccine										
	VicS-v vaccine										
^N1/08	Unknown (N1/62)	A3 vaccine	ns	6.94E-17	2.05E-10	8.35E-06	2.45E-05	1.41E-04	ns	ns	4.01E-09
N1/03	I vaccine	Q1/13	7.04E-14	ns	8.78E-07	7.36E-05	2.21E-05	ns	ns	ns	4.23E-08
	V5/90										
	VicS-v vaccine										
^Q1/73	N1/62	I vaccine	1.45E-10	ns	5.42E-09	7.05E-06	2.87E-05	2.57E-05	ns	ns	ns
Q1/73	N1/62	I vaccine	8.18E-11	ns	4.60E-09	1.12E-04	6.70E-05	ns	ns	ns	6.66E-08
	A3 vaccine	V5/90									
^Q1/73	A3 vaccine	I vaccine	5.68E-03	2.45E-03	ns	5.79E-09	4.79E-06	ns	ns	ns	1.20E-06
^N1/03	Q1/73	A3 vaccine	1.03E-02	8.42E-08	8.09E-03	1.20E-02	3.34E-05	1.03E-05	ns	ns	5.97E-05
	I vaccine										
	V5/90										
	VicS-v vaccine										
^N1/03	A3 vaccine	Unknown (N1/62)	3.26E-03	3.82E-10	3.34E-04	3.63E-08	7.05E-08	ns	ns	ns	1.49E-04
^N1/03	A3 vaccine	VicS-v vaccine	2.91E-05	4.21E-10	1.44E-05	1.42E-07	8.93E-08	ns	ns	ns	5.87E-07
		I vaccine									
		V5/90									

### Tracheal ciliostasis

The ciliostasis observations are summarized in Table 5. The proportion of chickens with tracheal ciliostasis was significantly higher in the groups infected with N1/62 (75%, *P*=0.02) or N1/03 (90%, *P*=0.002) than in the uninfected group (0%). The proportion of birds with ciliostasis was also significantly higher in the group infected with N1/03 than in the group infected with N1/08 (30%). The proportions of birds with ciliostasis did not differ between the uninfected control group and the groups infected with Q1/73 (56%, *P*=0.09), V5/90 (50%, *P*=0.10), N1/08 (30%, *P*=0.50) or Q1/13 (50%, *P*=0.10).

**Table 5.** Tracheal ciliostasis in birds infected with different strains of IBV at day 9 post-infection

	Ciliostasis		
Strain	Positive	Negative	Proportion positive (%)
Negative control	0	5	0^a^
N1/62	6	2	75^bc^
Q1/73	5	4	55^abc^
V5/90	5	5	50^abc^
N1/03	9	1	90^c^
N1/08	3	7	30^ab^
Q1/13	5	5	50^abc^

Proportions labelled with the same superscript letter did not differ significantly (Fisher’s exact test, *P* > 0.05).

### Histopathological changes in the trachea

#### Mucosal thickness

**Fig. 6.**
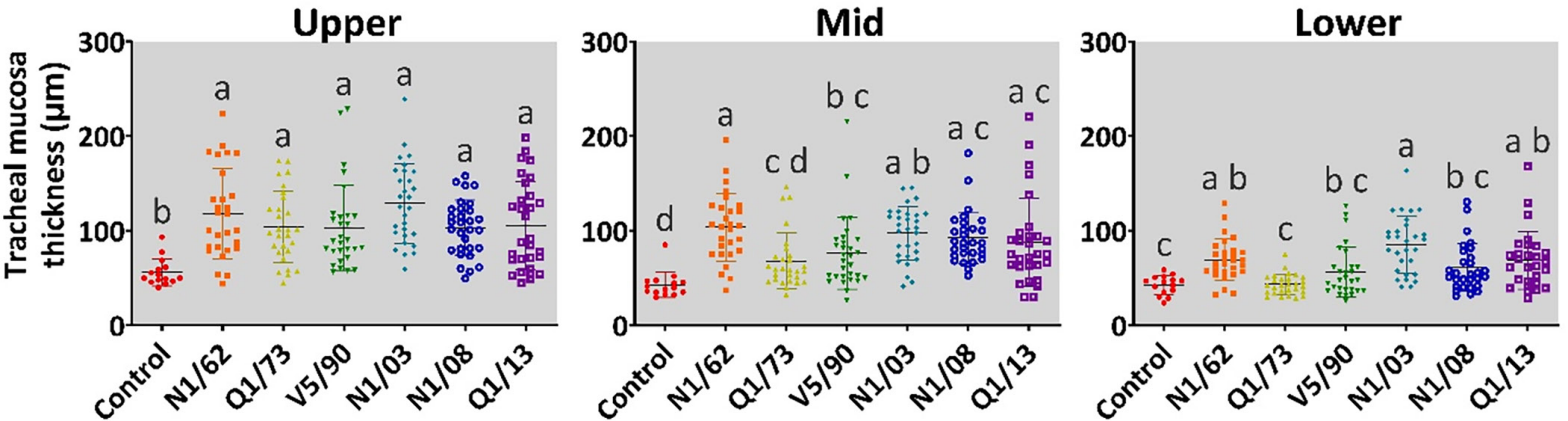
Thickness of the tracheal mucosae of chickens infected with different strains of IBV. The groups labelled with the same letter in the same graph did not differ significantly (ANOVA, *P*>0.05).

**Fig. 7.**
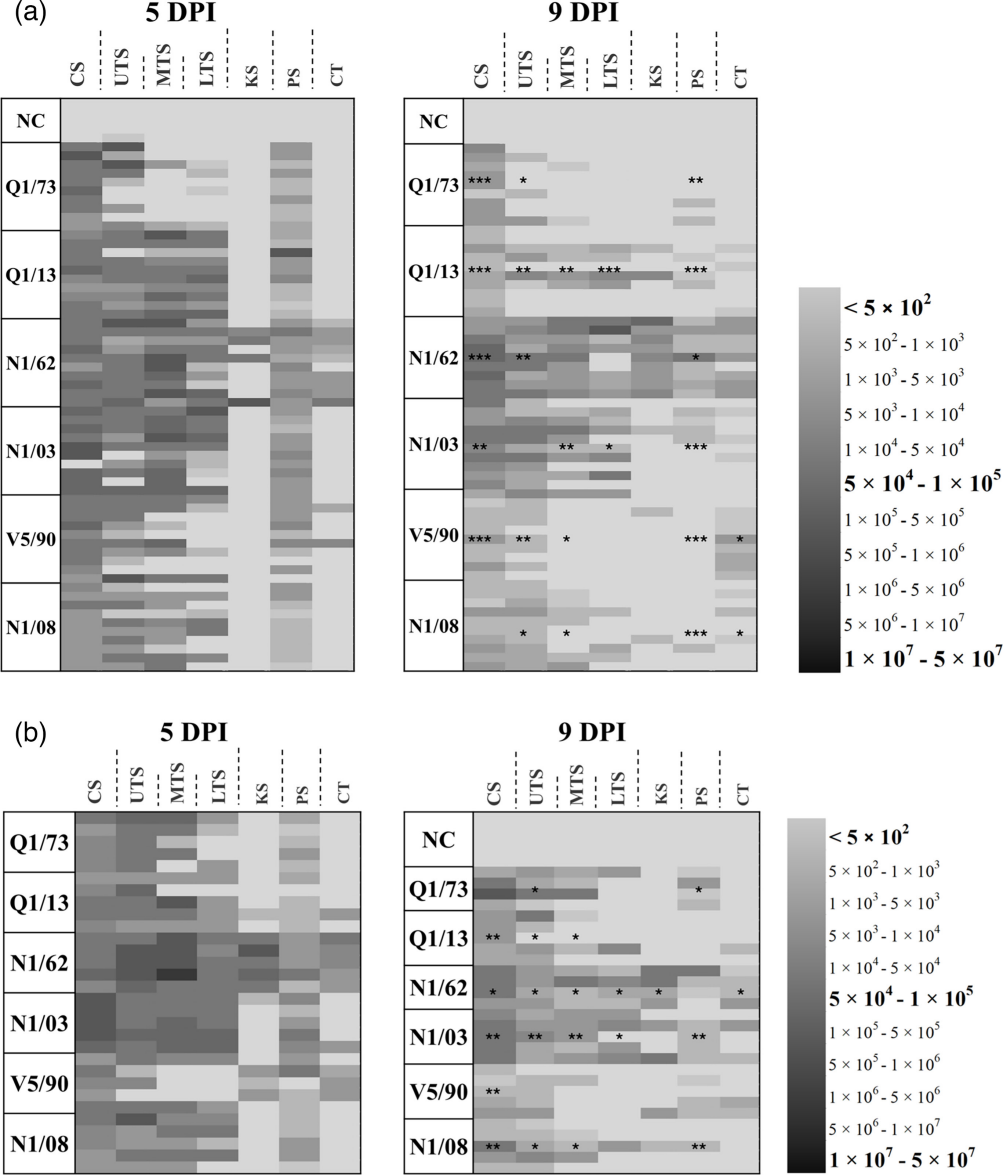
Heatmap showing the IBV genome concentrations in the tissues of (**a**) infected and (**b**) in-contact chickens. The IBV genome concentrations were determined by reverse transcriptase qPCR and normalized using a reverse transcriptase qPCR assay targeting the chGAPDH transcript as the reference. Each row represents the measurements for an individual chicken. Darker colours indicate a higher concentration of the IBV genome. The labels on the left show the IBV strain. CS, conjunctival swab; UTS, MTS and LTS, upper, mid and low tracheal swabs, respectively; KS, kidney swab; PS, proventricular swab; CT, caecal tonsils. NC, negative control. Asterisks indicate when the median IBV genome concentrations in each tissue for each strain differed significantly between day 5 and day 9 post-infection (Mann–Whitney; *, *P*<0.05; **, *P*<0.01; ***, *P*<0.001).

The mucosae of the upper, mid and lower tracheal sections of the birds in the uninfected control group appeared normal, except for some degree of degradation and sloughing of the epithelium in some sections that were interpreted as artefactual. In contrast, the mucosae of the trachea in most chickens infected with the different strains of IBV were altered. The mean tracheal mucosal thickness measurements are shown in Table 6 and Fig. 6. There was a significant difference between the mean mucosal thickness of the upper trachea of the uninfected control group and that of each of the groups infected with IBV. The strains that induced the greatest increase in the mean mucosal thickness of the upper trachea were N1/62 (118±48 µm) and N1/03 (129±42 µm), but these did not differ significantly from those of the birds infected with any of the other four strains. In the mid trachea, the mean mucosal thickness of the birds infected with strain Q1/73 did not differ from that of the uninfected chickens and was significantly less than that of the groups infected with N1/62 (*P*=0.002) or N1/03 (*P*=0.018). The mean mid-tracheal mucosal thicknesses of the birds infected with N1/62 (104±36 µm, *P*<0.0001), V5/90 (78±38, *P*=0.035), N1/03 (98±28 µm, *P*=0.0001), N1/08 (92±27 µm, *P*=0.0001) and Q1/13 (88±47 µm, *P*=0.0008) were significantly greater than that of the uninfected control group (43±13 µm). The differences between the infected and birds from the uninfected control group in the lower trachea were not as great as in the upper and mid trachea. The groups infected with N1/62 (70±22 µm, *P*=0.01), N1/03 (85±30 µm, *P*<0.0001) and Q1/13 (69±31 µm, *P*=0.02) had a mean lower tracheal mucosal thickness significantly greater than that of the uninfected control group. As shown in Table 6 and Fig. 7, IBV infection induced more severe inflammation, and there were higher levels of replication of IBV in the upper trachea than in the middle trachea, while there was a minimal impact on the lower trachea.

**Table 6.** Mean mucosal thickness measurements of the upper, mid and lower trachea in uninfected chickens and chickens infected with the different strains of IBV

	Mean mucosal thickness±standard deviation (µm)		
Strain	Upper	Mid	Lower
Negative control	56±14^ b,1^	43±13^ d,1^	43±10^ c,1^
N1/62	118±48^ a,1^	104±36^ a,1^	70±22^ ab,2^
Q1/73	104±38^ a,1^	68±30^ cd,2^	44±11^ c,3^
V5/90	103±45^ a,1^	78±38^ bc,2^	56±27^ bc,2^
N1/03	129±42^ a,1^	98±28^ ab,2^	85±30^ a,2^
N1/08	103±29^ a,1^	92±27^ ac,1^	61±25^ bc,2^
Q1/13	105±46^ a,1^	88±47^ ac,12^	69±31^ ab,2^

Values in the same column labelled with the same superscript letter did not differ significantly (*P*>0.05). Values in the same row labelled with the same superscript number did not differ significantly (*P*>0.05).

### Lesion grades

Tracheal and kidney lesion scores from both the infected and in-contact groups are shown in Tables S1 and S2.

On day five post-infection (Table 7), the median mid-tracheal lesion grades in the birds exclusively from the directly infected birds (excluding the in-contact) were high (between 1.8 and 2.3) in all groups except those infected with strain Q1/73 (0.5). In the in-contact birds, the grades were also high (between 2.0 and 3.0), except for those in contact with birds infected with strains Q1/73 or V5/90, which had median grades of 1.5 and 1.0, respectively. At 9 DPI, the median grade was significantly higher in the birds infected with strain N1/03 than in the uninfected control group. While the highest median lesion grade was seen in the birds infected with N1/62, this did not differ significantly from the median lesion grades in the other groups. The in-contact birds had similar grades to those of the inoculated birds, with the highest median grades seen in those birds in contact with birds infected with strains N1/62 or N1/03 (median grade of 2), although the median grades did not differ significantly between the groups. Six birds in the uninfected control group had some sloughing and congestion of the epithelium, like that seen in acute infections with IBV, with grades of 2 or 3 at day 5 and grades of 1 or 3 at day 9 post-infection. However, eight birds in the uninfected control group had no detectable lesions (grades of 0). Only the birds infected with strain N1/03 had a significantly higher median tracheal grade than the uninfected control group (*P*=0.022). However, this did not differ from the median tracheal lesion grades for the other groups of infected birds.

**Table 7.** Histopathological lesion grades for the mid-tracheal sections from uninfected chickens and chickens infected with different strains of IBV at days 5 and 9 post-infection

	Median histopathological lesion grade (range)			
	Day 5 post-infection		Day 9 post-infection	
Strain	Infected	In-contact	Infected	In-contact
Negative control	2.0 (0–3)^a,b^	2.0 (0–3)^a^	0.0 (0–3)^a^	0.0 (0–3)^a^
N1/62	1.8 (1–4)^a,b^	3.0 (1.5–4)^a^	2.3 (1–4)^a^,^b^	2.0 (1–3)^a^
Q1/73	0.5(0.5–4)^b^	1.5 (0–3)^a^	1.0 (0–4)^a^,^b^	1.5 (1–2)^a^
V5/90	1.8 (0–3.5)^a,b^	1.0 (0–2)^a^	1.0 (0–3)^a^,^b^	1.0 (0–3)^a^
N1/03	2.3 (0.5–3)^a,b^	2.0 (0–4)^a^	1.5 (1–3)^b^	2.0 (1.5–4)^a^
N1/08	2.0 (2–4)^a^	3.0 (1–4)^a^	1.3 (0–2.5)^a^,^b^	1.0 (0.5–2.5)^a^
Q1/13	1.8 (1–4)^a,b^	2.0 (0.5–3)^a^	1.0 (0–1.5)^a^	1.5 (0–2.5)^a^

Values in the same column labelled with the same superscript letter did not differ significantly (Kruskal–Wallis test, false discovery rate corrected, *P* > 0.05).

### Histopathological changes in the kidneys

The primary histopathological change observed in the kidneys was lymphocytic interstitial infiltration; therefore, lesion grading was based solely on this feature. Other histopathological changes seen in the kidneys of birds infected with IBV in previous studies, such as tubular degeneration and urate accumulation, were not seen in any of the birds in this study. As shown in Table 8, the highest renal lesion grades were seen in the chickens infected with strain N1/62. In the in-contact chickens exposed to strain N1/62, the renal lesion grades ranged from 0 to 3 at 5 DPI and from 2 to 3 at 9 DPI. In each of the groups infected with the other strains, there were a small number of birds with renal lesions with grades of 1 or 2. One bird in the uninfected control group had a renal lesion grade of 1 at 5 DPI, and one bird from the uninfected control group had a renal lesion grade of 1 at 9 DPI.

**Table 8.** Histopathological renal lesion grades of uninfected chickens and chickens infected with different strains of IBV at days 5 and 9 post-infection

	Median histopathological renal lesion grade (range)			
	Day 5 post-infection		Day 9 post-infection	
Strain	Infected	In-contact	Infected	In-contact
Negative control	0 (0–1)^a^	0 (0–1)^a^	0 (0–1)^b****^	0.0 (0–1)^b****^
N1/62	0 (0–3)^a^	3 (0–3)^a^	3 (2–3)^a^	2.5 (2–3)^a^
Q1/73	0 (0–1)^a^	0 (0–0)^a^	0 (0–0)^b****^	0.0 (0–1)^b**^
V5/90	0 (0–0)^a^	0 (0–0)^a^	0 (0–2)^b****^	0.0 (0–2) ^b**^
N1/03	0 (0–1)^a^	0 (0–1)^a^	0 (0–1)^b****^	0.0 (0–1) ^b***^
N1/08	0 (0–1)^a^	0 (0–2)^a^	0 (0–0)^b****^	0.0 (0–0) ^b****^
Q1/13	0 (0–0)^a^	0 (0–0)^a^	0 (0–2) ^b****^	0.0 (0–0) ^b****^

Values in the same column labelled with the same superscript letter did not differ significantly (Kruskal–Wallis, false discovery rate corrected, *P*>0.05). The asterisks indicate the level of significance (*, *P*<0.05; **, *P*<0.01; ***, *P*<0.001; ****, *P*<0.0001).

### IBV genome concentrations in tissues

All the results are presented in this section as IBV genome concentrations are referred to IBV genome copies for every 10^5^ copies of the housekeeping gene chGAPDH. All results will be divided into two sections: infected, which are the groups that directly received a dose of one of the strains of IBV used in the present study, and in-contact, which are the chickens in each group that did not receive a direct inoculation of any IBV strain, but were incorporated into the same isolators with previously infected chickens.

### Conjunctiva

Results are presented in Figs7  8. *Infected*: The genome copies on the conjunctival swabs from the infected chickens did not differ between strains at 5 DPI, and all of the infected groups had significantly higher IBV genome concentrations compared with the uninfected control group. There was a marked decrease in viral genome copies at 9 DPI for strains V5/90 (98.5%), Q1/13 (96%), N1/03 (95.8%) and Q1/73 (95.6%) and a more moderate decrease for strain N1/62(84.4%). The decrease was not as marked as in the other groups for the strain N1/08 (34.7%), as its replication rate was near 1-log lower at 5 DPI compared with the other groups. *In-contact*: In the in-contact group, there were significant diﬀerences between the strains at 5 DPI. Some strains presented fewer IBV genome copies, 2.3, 3.5 and 5.7×10^6^ for V5/90, N1/08 and Q1/13, respectively, compared with 1.4, 1.6 and 4.2×10^7^ for Q1/73, N1/62 and N1/03, respectively. The decrease in genome concentrations at 9 DPI was relatively high in most strains, being of 97%, 96.8%, 93.5%, 91.7% and 91.5% for strains Q1/13, N1/03, N1/08, N1/62 and V5/90, and more moderate in those infected with Q1/73, with a reduction of 81.7%.

**Fig. 8.**
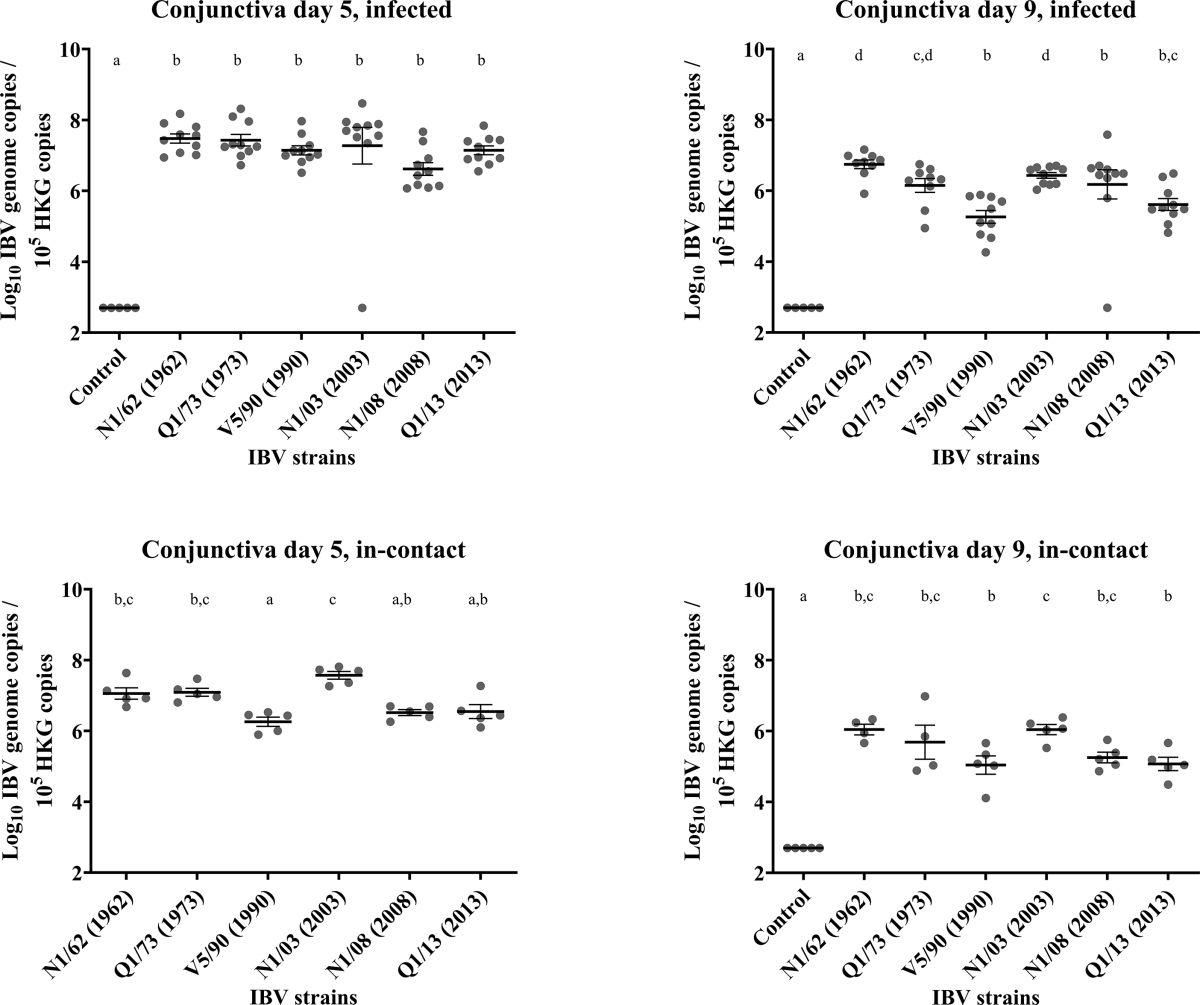
Number of copies of the IBV genome found in the conjunctival tissue samples collected by swab from chickens infected with different strains of IBV. The numbers are relative to the number of copies of the reference or ‘housekeeping’ gene found in the same samples. The bars indicate the mean and the sem. Limit of detection of the qPCR: 5×10^2^ IBV genome copies. The groups labelled with the same letter in the same graph did not differ significantly (ANOVA, *P*>0.05). HKG, housekeeping gene.

### Upper trachea

Results are presented in Figs7  9. *Infected*: At 5 DPI, the IBV genome concentrations in the upper trachea were high (between 8.1×10^6^ to 3.7×10^7^) in most of the birds in each of the infected groups, and there was no significant difference in the concentrations between them. All the samples (10/10) from the chickens infected with N1/62 were positive to IBV, while 7/10, 9/10, 8/10, 9/10 and 9/10 were positive from the chickens infected with Q1/73, V5/90, N1/03, N1/08 and Q1/13, respectively. At 9 DPI, the IBV genome concentrations had decreased in the upper trachea. The genome concentrations at 9 DPI were higher in the chickens infected with the strains N1/62 (1.1×10^6^), N1/03 (3.3×10^6^) and N1/08 (3.1×10^6^), with all the chickens testing positive, than in the chickens infected with strains Q1/73 (1.0×10^5^, 3/10 positive) and V5/90 (7.4×10^5^, 6/10 positive). The mean genome concentrations were also relatively high for Q1/13 (6.9×10^5^), but with only 4/10 samples positive. *In-contact*: In this group, the replication rate in all groups was as high as in those directly infected (1.1×10^7^ to 2.7×10^8^), with only one bird being negative in the V5/90 group. There was also a decrease in the IBV genome concentrations at day 9 DPI in all groups (1.0×10^5^ to 5.7×10^5^), and there was no signiﬁcant diﬀerence between the groups, with 2/5 and 4/5 samples positive in the V5/90 and Q1/13 groups.

**Fig. 9.**
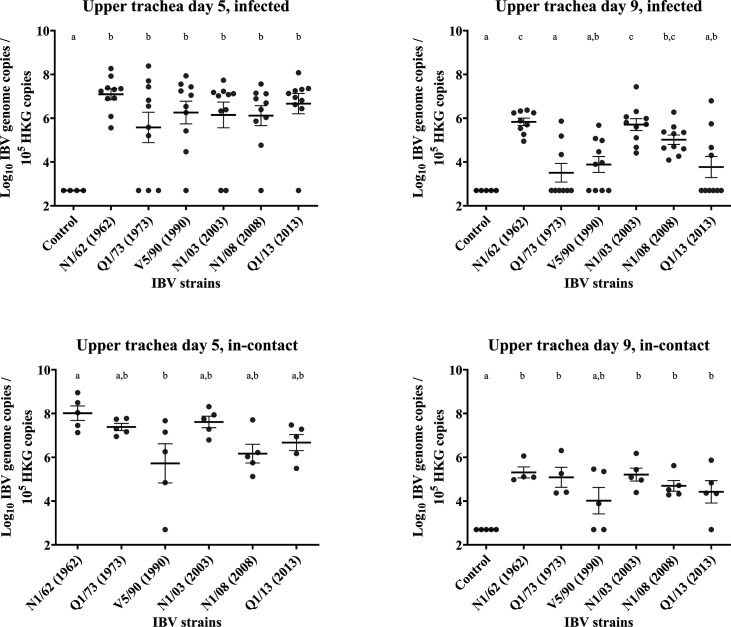
Number of copies of the IBV genome found in the upper trachea tissue samples collected by swab from chickens infected with different strains of IBV. The numbers are relative to the number of copies of the reference or ‘housekeeping’ gene found in the same samples. The bars indicate the mean and the sem. Limit of detection of the qPCR: 5×10^2^ IBV genome copies. The groups labelled with the same letter in the same graph did not differ significantly (ANOVA, *P*>0.05). HKG, housekeeping gene.

### Mid trachea

Results are presented in Figs7  10. *Infected*: The IBV genome concentrations in the mid trachea were as high as in the conjunctival and upper tracheal swabs (>1.6×10^6^), except for those in the group infected with Q1/73, with mean IBV genome concentrations being significantly lower than the other groups (3.4×10^4^, *P*<0.05). The IBV genome concentrations were significantly higher in the chickens infected with N1/62 (8.5×10^7^) than in those infected with Q1/73 (3.4×10^4^, *P*<0.0001) and V5/90 (2.5×10^7^, *P*=0.03). There were also higher levels in those infected with N1/03 (6.9×10^7^) compared with those infected with Q1/73 (*P*<0.0001) and V5/90 (2.5×10^7^, *P*=0.02), than in those infected with Q1/73 (3.4×10^4^, *P*<0.0001) and V5/90 (2.5×10^7^, *P*=0.03), but not significantly higher compared with the chickens from groups N1/08 (5.6×10^6^) and Q1/13 (7.3×10^7^). On the other hand, the genome concentrations observed in the Q1/73 group did not differ significantly from those in the uninfected control group (*P*=0.99). The concentration of the IBV genome in the mid trachea was lower at 9 DPI in all groups, with the highest concentrations detected in the birds infected with N1/62 (1.5×10^7^), which were slightly lower than the concentrations found in the same group at 5 DPI (8.5×10^7^). A similar situation occurred with the group N1/03, with the IBV genome concentrations decreasing slightly from 6.8 to 5.2×10^7^. All the other groups, except for Q1/73, V5/90, N1/08 and Q1/13, had a reduction between 88.6–97.1%. In these groups, only 1/10, 4/10 and 4/10 were positive to IBV, respectively. In the Q1/73 group, as it happened at 5 DPI, only two chickens were positive for IBV. *In-contact*: At 5 DPI, the only group with genome concentrations significantly higher than the rest of the other groups was N1/62, with IBV genome concentrations that were even higher than those in the infected groups described above (5.6×10^8^). Interestingly, the proportion of positives in the group Q1/73 was higher in the in-contacts (4/5) than in the infected (1/5). The group with the lower IBV genome concentrations was V5/90, with 2/5 positive samples. The reduction in IBV genome concentrations at 9 DPI was marked in all groups. Interestingly, the group with less reduction in IBV genome concentrations was Q1/73 (54.0%), when the other groups had a reduction of 98.0–99.4%. Among these groups, V5/90 had the lowest reduction (98.0%), but also the lowest replication, as only 1/5 samples were negative, and the positive samples obtained an IBV genome concentration of only 8.8×10^4^.

**Fig. 10.**
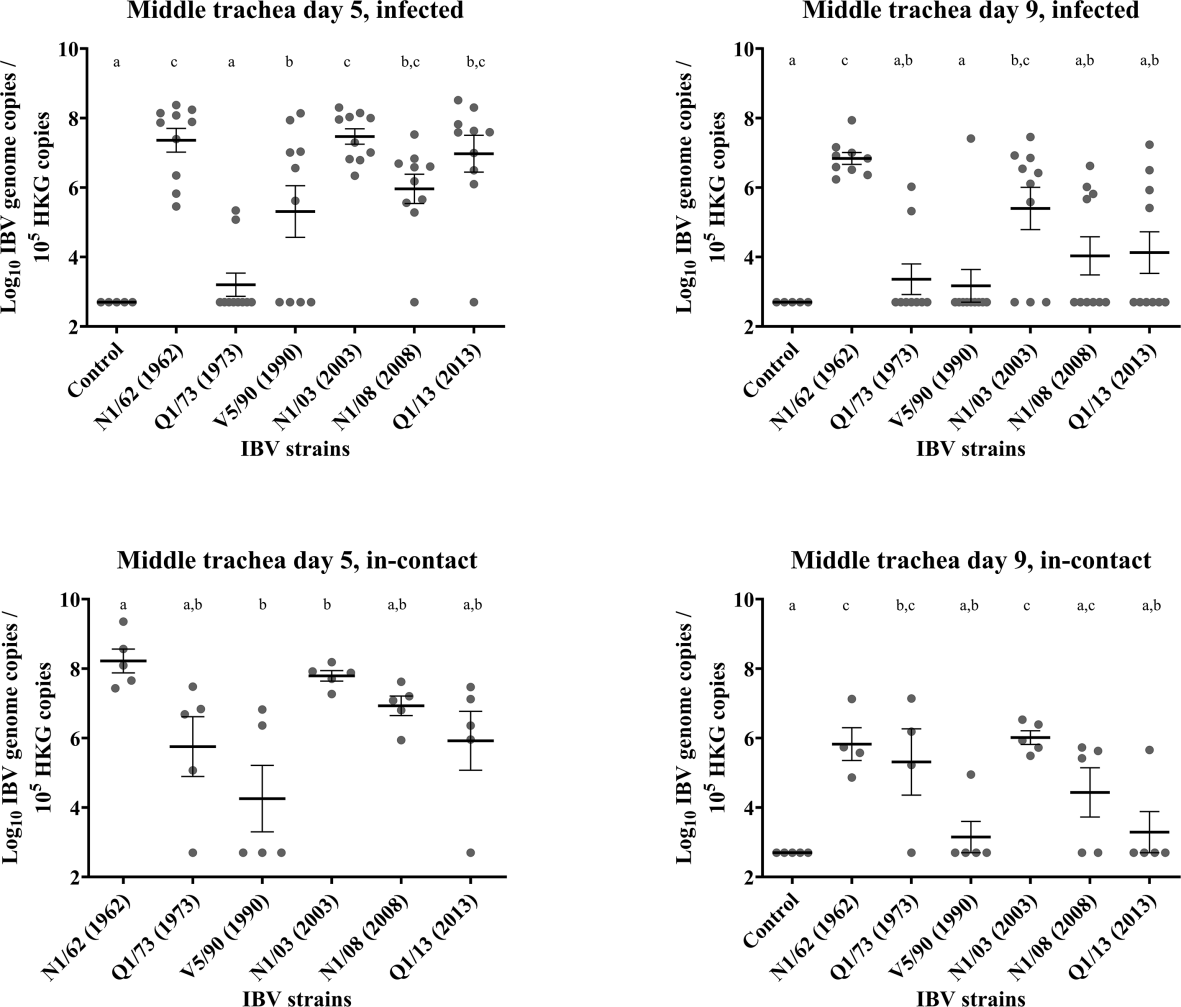
Number of copies of the IBV genome found in the middle trachea tissue samples collected by swab from chickens infected with different strains of IBV. The numbers are relative to the number of copies of the reference or ‘housekeeping’ gene found in the same samples. The bars indicate the mean and the sem. Limit of detection of the qPCR: 5×10^2^ IBV genome copies. The groups labelled with the same letter in the same graph did not differ significantly (ANOVA, *P*>0.05). HKG, housekeeping gene.

### Lower trachea

Results are presented in Figs7  11. *Infected*: At 5 DPI, birds infected with N1/62, N1/03 or Q1/13 had significantly higher concentrations of IBV genome in the lower trachea (1.1, 1.4 and 1.2×10^8^, respectively) compared with the rest of the groups. The lowest concentrations of IBV genome were found in the birds from groups Q1/73, V5/90 and N1/08, with only 3/10, 4/10 and 5/9 individuals positive, and their IBV genome concentrations were not significantly higher than those found in the uninfected control group. Only those from the V5/90 group exhibited IBV genome concentrations significantly lower than those from the N1/62, N1/03 and Q1/13 groups. At 9 DPI, the IBV genome concentrations drastically decreased in all groups, with a reduction of 99.7% in the Q1/73, 94.5% in N1/03, 99.6% in N1/08 and 96.7% in Q1/13 groups. The reduction was lower in the V5/90 group, with 64.9%, and it was only 41.7% for those in the N1/62 group. In the V5/90 group, only one chicken was a strong positive at 9 DPI, while the rest of the chickens in the group were negative, which increased the mean value and explained the lower reduction. *In-contact*: At 5 DPI, the birds from the N1/03 group had the highest IBV genome concentrations, with an average of 6.1×10^8^ genome copies. The only group that had a significantly lower number of IBV genome concentration compared with the N1/03 group was those from the V5/90 group. The only groups where all individuals were positive at 9 DPI were N1/62 and N1/03. N1/03 had IBV genome concentrations significantly higher than all groups, except for N1/62 (6.4×10^8^). However, the reduction in IBV genome concentrations and number of positives was drastically reduced in all groups. In the Q1/73, N1/08 and Q1/13 groups, only 1/5 chickens were positive, while in group V5/90, all chickens were negative. The reduction in genome concentrations was more marked in the groups N1/62, V5/90, N1/03 and N1/08, with reductions of 97.6%, 99.99%, 99.0% and 97.3%, respectively. The reduction in IBV genome concentration was only 44.8% in the Q1/73 group, but only 1/5 chickens were positive. A similar situation occurred with those from the Q1/13 group, where the reduction in IBV genome concentrations was 71.4%, but with only 1/5 individuals positive to IBV at 9 DPI.

**Fig. 11.**
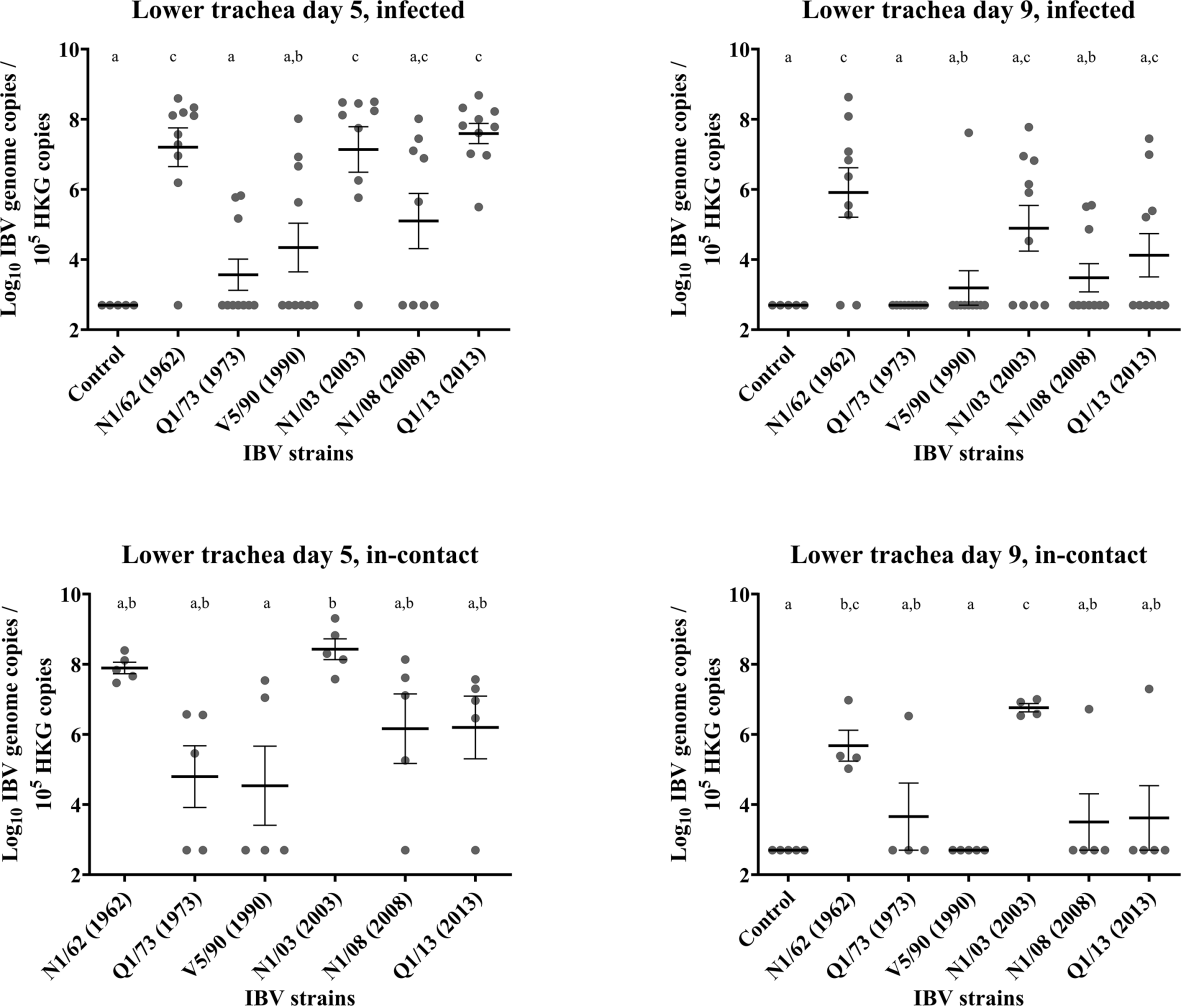
Number of copies of the IBV genome found in the lower trachea tissue samples collected by swab from chickens infected with different strains of IBV. The numbers are relative to the number of copies of the reference or ‘housekeeping’ gene found in the same samples. The bars indicate the mean and the sem. Limit of detection of the qPCR: 5×10^2^ IBV genome copies. The groups labelled with the same letter in the same graph did not differ significantly (ANOVA, *P*>0.05). HKG, housekeeping gene.

### Kidney

Results are presented in Figs7  12. *Infected:* At 5 DPI, the only kidney samples with a relatively high concentration of IBV genome were from those birds infected with N1/62 (1.4×10^6^), while in the other groups, all birds remained negative. Interestingly, at 9 DPI, even though the average genome concentrations appear similar in those infected with N1/62 (1.1×10^5^), the number of positive individuals increased from 4/10 at 5 DPI to 9/9 at 9 DPI; one chicken in this N1/62 infected group died at 7 DPI. The kidney lesions in this dead bird were coincidental with those described for nephrotic IBV (Fig. S1). The kidneys were pale and had exaggerated tubular patterns due to deposition of uric acid. *In-contact:* IBV RNA was detected in in-contact birds exposed to strains V5/90, N1/03, N1/08 or Q1/13, with 1/10 individuals positive from the first three groups and 2/10 positives in the Q1/13 group. However, the mean genome concentrations detected (between 5.0×10^2^ and 1.1×10^4^) did not diﬀer signiﬁcantly from that of the uninfected control group. In these chickens, the replication in the kidney appeared more prematurely compared to that in the infected groups. At 5 DPI, all individuals (5/5) were positive to IBV in those birds from the N1/62 group, with an average IBV genome concentration of 3.7×10^6^, which was significantly higher compared with all the other groups (*P*<0.0001). As in the infected birds at 9 DPI, the chickens that were in contact with Q1/73 were all negative for IBV, but also those in contact with N1/08. In the groups V5/90, N1/03 and Q1/13, 3/5, 1/5 and 1/5 individuals were positive to IBV, respectively. At 9 DPI, there was a marked reduction in IBV genome concentrations in those in contact with N1/62 (2.4×10^5^), although all individuals in that group remained positive. As it occurred at 5 DPI, all individuals were negative in the group Q1/73, but also in those from the Q1/13 group. In groups V5/90, N1/03 and N1/08, 1/5, 2/5 and 1/5 individuals were positive, respectively. All these groups, except for group N1/03, had significantly lower IBV genome concentrations compared with those in the N1/62 group.

**Fig. 12.**
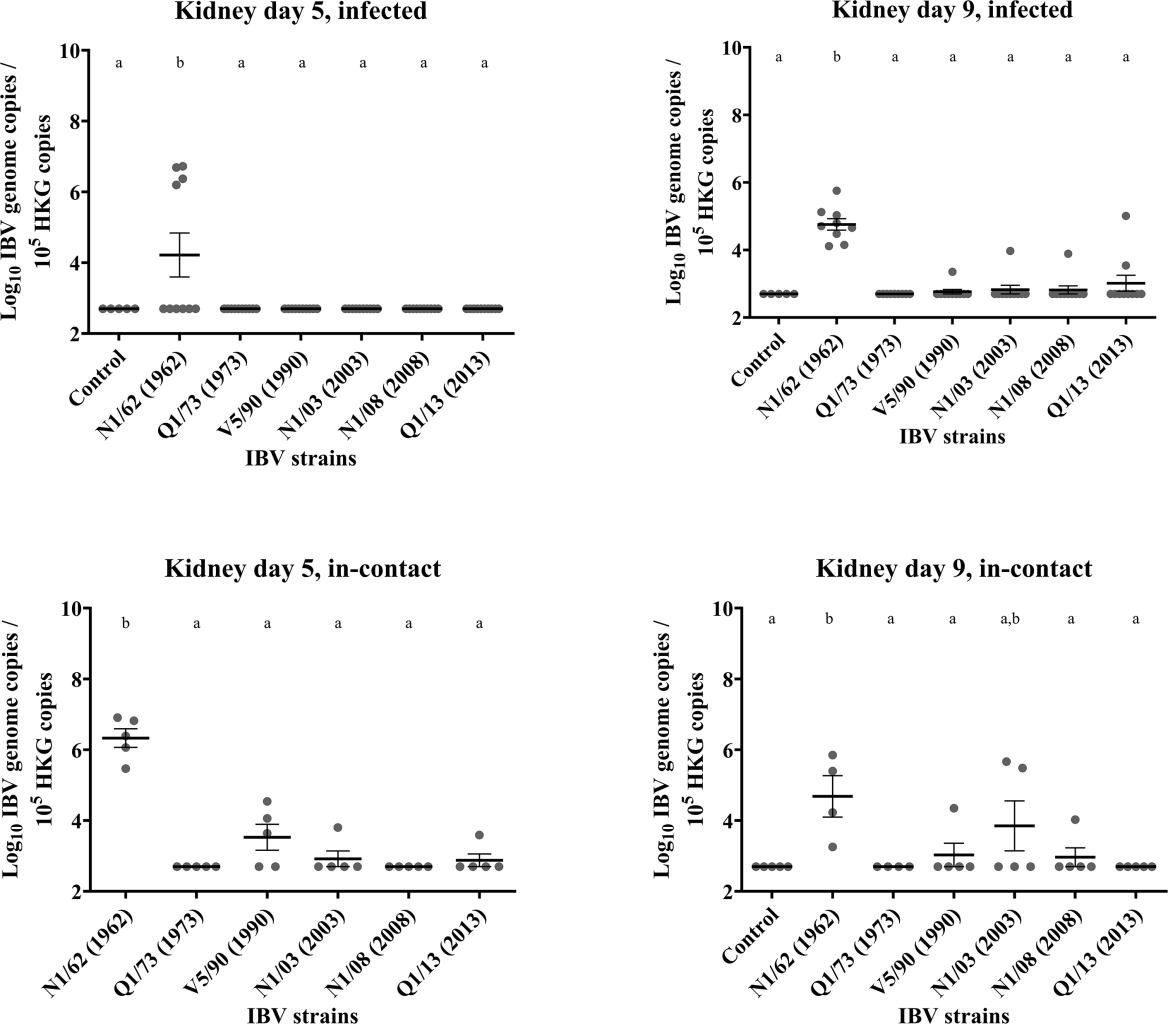
Number of copies of the IBV genome found in the kidney tissue samples collected by swab from chickens infected with different strains of IBV. The numbers are relative to the number of copies of the reference or ‘housekeeping’ gene found in the same samples. The bars indicate the mean and the sem. Limit of detection of the qPCR: 5×10^2^ IBV genome copies. The groups labelled with the same letter in the same graph did not differ significantly (ANOVA, *P*>0.05). HKG, housekeeping gene.

### Proventriculus

Results are presented in Figs7  13. *Infected:* The concentrations of IBV genome detected in the proventricular swabs of birds exposed to the six IBV strains included in this study were relatively high at 5 DPI, with almost all individuals being positive to IBV except for 2/10 in the V5/90 group. There were no significant differences in IBV genome concentrations between groups, and all of them had significantly higher IBV genome concentrations than those from the uninfected control group. However, at 9 DPI, the IBV genome concentrations remained high only for the birds infected with N1/62 (from 7.2×10^6^ at 5 DPI to 5.4×10^6^ at 9 DPI, a decrease of 24.3%), but had a marked decrease in all the other groups of birds by 64.7% in Q1/73 (only 1/10 individual positive), 99.9% in V5/90 (all negative), 93.65% in N1/03(4/10 individuals remained positive), 99.2% in N1/08(1/10 remained positive) and 99.1% in Q1/13(2/10 remained positive). *In-contact*: In the in-contact birds, the genome concentration at 5 DPI was still high, with only two individuals, 1/5 in group V5/90 and 1/5 in the group Q1/13, negative to IBV. Interestingly, at 9 DPI, the birds exposed to N1/62, Q1/73 and N1/03 still had IBV genome concentrations significantly higher than the uninfected control group, with 3/4, 4/4 and 4/5 individuals with detectable levels of the IBV genome. In the birds exposed to V5/90 and N1/08, only 2/5 and 1/5 individuals remained positive, while those from the Q1/13 were all negative, with mean genome concentrations that were not signiﬁcantly diﬀerent from those found in the uninfected control group.

**Fig. 13.**
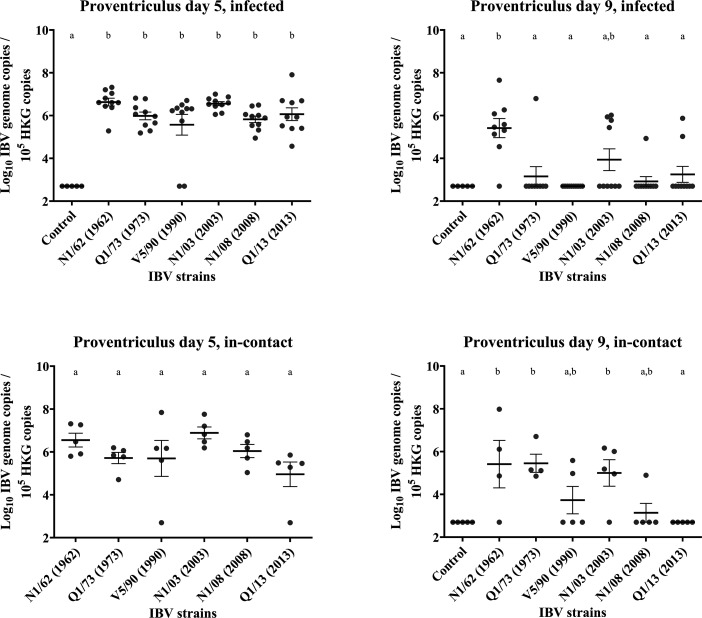
Number of copies of the IBV genome found in the proventricular tissue samples collected by swab from chickens infected with different strains of IBV. The numbers are relative to the number of copies of the reference or ‘housekeeping’ gene found in the same samples. The bars indicate the mean and the sem. Limit of detection of the qPCR: 5×10^2^ IBV genome copies. The groups labelled with the same letter in the same graph did not differ significantly (ANOVA, *P*>0.05). HKG, housekeeping gene.

### Caecal tonsils

Results are presented in Figs7  14. *Infected*: At 5 DPI, the IBV genome concentrations detected in the caecal tonsil were relatively low compared to other tissues examined, except the kidneys. Among the infected groups, the only group with a mean IBV genome concentration signiﬁcantly greater than the uninfected control group was the one infected with N1/62 (*P*<0.0001), and 2/10 birds in the group infected with V5/90 were positive at 5 DPI, but their mean genome concentrations did not differ from those in the uninfected control group (*P*=0.83). In the other groups, all chickens were negative. However, there was a marked increase of IBV genome concentrations at 9 DPI, particularly in those chickens from groups V5/90 (8/10 positive), N1/03 (3/10 positives), N1/08 (3/10 positives) and Q1/13 (3/10 positives). *In-contact:* The in-contact birds exposed to N1/62 had higher concentrations (3.2×10^6^, all positive) of IBV genome in their caecal tonsils compared to all the other groups, except for the V5/90 group (8.2×10^5^, but only 3/5 positive). In groups N1/03 (3.1×10^5^) and Q1/13 (1.1×10^5^), 2/5 and 1/5 individuals were positive to IBV, and all chickens remained negative in the Q1/73 and N1/08 groups. At 9 DPI, only the birds infected with the strain N1/62 remained all positive to IBV (3.3×10^5^), with a reduction of the IBV genome concentration of 89.6%. In the groups infected with V5/90 (9.8×10^4^), N1/03 (2.4×10^5^) and Q1/13 (7.3×10^4^), 2/5, 2/5 and 1/5 individuals were positive, but their mean IBV genome concentrations did not differ from those of the uninfected control group.

**Fig. 14.**
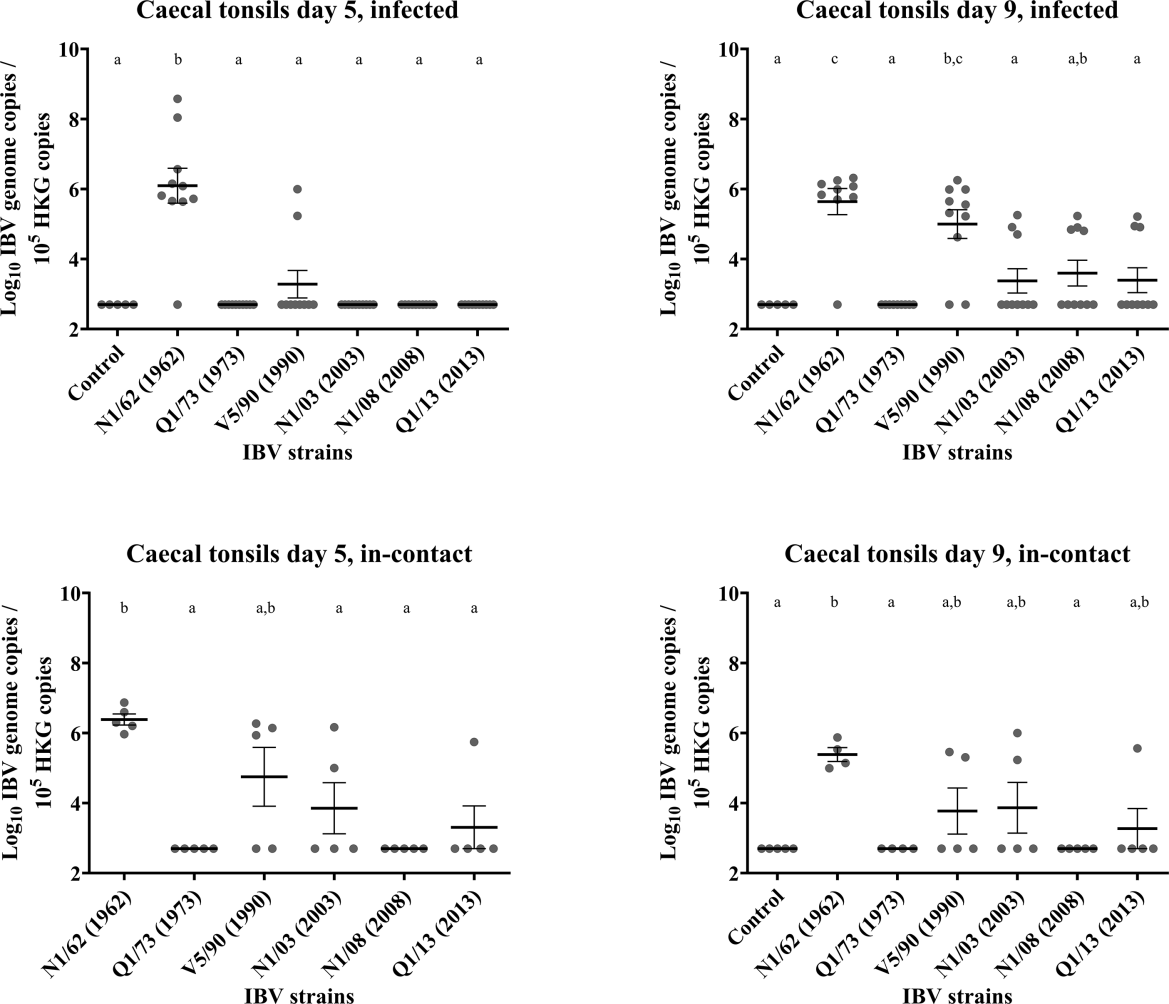
Number of copies of the IBV genome found in the caecal tonsil tissue samples collected by swab from chickens infected with different strains of IBV. The numbers are relative to the number of copies of the reference or ‘housekeeping’ gene found in the same samples. The bars indicate the mean and the sem. Limit of detection of the qPCR: 5×10^2^ IBV genome copies. The groups labelled with the same letter in the same graph did not differ significantly (ANOVA, *P*>0.05). HKG, housekeeping gene.

## Discussion

This is the first study to characterize the infection outcomes of the most recently isolated Australian IBV strains in chickens (twenty-first century) and, importantly, to compare these effects with those caused by older, more traditional strains, like N1/62, broadly known as the nephropathogenic T-strain. By the time this study was conducted, Q1/13 was the most recent isolate available. Unlike other countries such as the USA [25], Canada [26] or South Korea [27], Australia does not have a formal IBV surveillance programme, which explains the absence of more recent isolates. Additionally, the present study is the first study to classify the local strains using the most recent nomenclature. In the present study, it was shown that the infection with some of the most recent isolates of IBV caused renal lesions, but these were significantly less severe than those induced by the archetypal nephrotic strain of IBV, N1/62. Notably, all three recombinant strains isolated over the last 20 years caused severe respiratory disease, with one strain, N1/03, causing the most severe lesions and reaching the highest concentration in the lower trachea of all the strains assessed. A previous study [13] demonstrated that some of these most recent isolates (N1/03 and N1/08) emerged as a result of recombination, with at least one IBV vaccine strain (Armidale or VicS) as a major or minor parent. Thus, it appears that recombination between field strains and vaccine strains of IBV has yielded recombinant viruses with enhanced levels of virulence for the respiratory tract.

The levels of pathogenicity of strains N1/62 and Q1/73 in this study differed from those seen in previous studies. In a study by Ignjatovic *et al*. [17], chickens infected with N1/62 or Q1/73 strains had a high mortality rate. Q1/73 was presumed to replicate in the kidneys, as the chickens infected with this strain had mild clinical signs that may have been indicative of nephrosis syndrome, consisting of ‘hunched posture, depression, reluctance to move, emaciation, diarrhoea and a soiled vent’ [17]. Even though no gross lesions were detected in the kidneys of birds infected with this strain in our study, mild histopathological changes were detected, and the virus was also detected in kidney tissue. One possible explanation for the differences seen between the two studies could be the method used to titrate the inocula. Even though the chickens in both studies were inoculated with a dose of 10^4^ median infectious doses, by the same route (intraocular) and at the same age (2 weeks), in the study conducted by Ignjatovic *et al*. [17]*,* viral titres were determined by inoculating tracheal organ cultures and monitoring them for ciliostasis to calculate the median ciliostasis dose or CD_50_. In contrast, our study determined the viral titres by inoculating embryos and examining them for pathological changes (EID_50_), which may have higher sensitivity. Thus, the doses of actual viable viruses delivered to each bird in the study by Ignjatovic *et al*. [17] were potentially higher than in our study. The other possible explanation is a difference in the strains of chickens used in the two studies. In the study of Ignjatovic *et al*. [17], the SPF chickens used were an inbred line previously found to be very susceptible to renal disease induced by infection with N1/62. The lesions in the trachea of chickens inoculated with strain N1/08 in our study were very similar to those observed in previous studies [18], with a mild increase in the thickness of the lamina propria induced by lymphocytic infiltration and pathological changes in both the upper and lower trachea.

One widely used method to assess IBV pathogenicity is measuring ciliary activity in the trachea of infected birds [28,33]. This method is also used to evaluate the protection provided by vaccines against IBV challenge [30,34,36]. The study described here found that the proportion of birds in which complete ciliostasis was induced differed between the IBV strains, from moderate (N1/08) to high (N1/03). The proportion of birds with ciliostasis was significantly higher in the groups infected with strains N1/62 and N1/03 than in the uninfected control group, suggesting that these two strains had a higher level of pathogenicity for the respiratory tract than the other four strains examined. This conclusion is supported by the differences seen between the histopathological changes in the tracheal mucosa by the different groups of birds, as these two strains induced the greatest increases in the thickness of the mucosa in the upper, mid and lower trachea, even though all strains induced an increase in the mucosal thickness in the upper trachea. Importantly, minimal histopathological changes were observed in the lower trachea, except in birds infected with strain N1/03. A limitation of this study is that only a single tracheal section was assessed for ciliostasis. Future studies with a more comprehensive evaluation of multiple tracheal segments would be needed to fully determine potential ciliostasis effects.

The differences in the concentrations of IBV genomes between the strains in different tissues at 5 and 9 DPI indicated that infection with strains N1/62 and N1/03 persisted for a longer period and affected a wider range of organs. It is notable that all the IBV strains tested in the present experiment replicated in the proventriculus, even though they did not cause any detectable lesions (such as proventriculitis), as has been seen with Chinese strains (Yu *et al*. [37] and the QX-like strain isolated in the UK (Ganapathy *et al*. [38]. Also, some of the stains used in the present study replicated in the kidney but did not cause any degree of histological changes or clinical signs in the chickens. The viral doses used to inoculate chickens in this study were like those used in both earlier studies. This demonstrates that these viruses can replicate in some organs without causing apparent damage.

It was also observed that, although all strains used in this study were able to actively replicate in the upper trachea, only N1/62, N1/03, N1/08 and Q1/13 achieved replication in the lower trachea, in contrast to the poor replication of Q1/73 and V5/90 in the same region. This indicates that the more recent isolates (2003, 2008 and 2013) have evolved and become better adapted to the respiratory tract than the older strains included in this study (1973 and 1990). Interestingly, N1/62, despite belonging to the older ‘traditional’ group (1962), also replicated efficiently in the lower trachea, reflecting its high pathogenicity.

In conclusion, this study demonstrates that the more recent isolates appear as recombinant strains, with vaccine strains serving as major or minor parental sequences. These recombinant IBVs (all isolated during the twenty-first century) have acquired stronger tropism and greater virulence for the respiratory tract, characterized by more persistent replication in the trachea and more severe lesions in the lower respiratory tract. It confirms that IBV produces early replication in the upper respiratory tract, which declines rapidly after 1 week of infection. On the contrary, some tissues, including the caecal tonsils and kidneys, exhibited a more delayed replication, increasing from 5 to 9 DPI. It has also demonstrated the capacity of multiple strains of IBV to replicate to significant levels in the proventriculus and kidneys, even though they do not cause detectable proventriculitis or nephritis, respectively. However, the immunological reasons behind these differences remain unknown and might be the subject of future investigations. This study also highlights the need for a formal IBV surveillance programme in Australia, which would facilitate research on the efficacy of the currently available commercial vaccines.

## Supplementary material

10.1099/jgv.0.002213 video 1.

10.1099/jgv.0.002213 video 2.

10.1099/jgv.0.002213 Uncited Supplementary Material 1.

## Abbreviations

AF: allantoic fluid
CD: ciliostatic doses
chGAPDH: chicken glyceraldehyde 3-phosphate dehydrogenase
DPC: days post-contact
DPI: day post-inoculation
EID: embryo infectious doses
IBV: infectious bronchitis virus
qPCR: quantitative PCR
RS: reference strain
SPF: specific-pathogen-free
VTM: viral transport medium
