# Supplementary material for: Australian strains of the avian coronavirus infectious bronchitis virus predominantly target the respiratory tract rather than the kidneys in specific-pathogen-free chickens
Source: J Gen Virol. 2026 Jan 23;107(1):002213. doi: 10.1099/jgv.0.002213 (PMC12829727; doi:10.1099/jgv.0.002213)
Supplement: Uncited Supplementary Material 1. [file jgv-107-02213-s003.pdf]

Supplementary material

Table S1. Mid tracheal lesion grades for each bird from both the infected and in-contact group, with each strain used in the study and at each timepoint after infection/mock infection.

	N1/62		Q1/73		V5/90		N1/03		N1/08		Q1/13		Control	
	5 DPI	9 DPI	5 DPI	9 DPI	5 DPI	9 DPI	5 DPI	9 DPI	5 DPI	9 DPI	5 DPI	9 DPI	5 DPI	9 DPI
**No lesions**			0.0	0.0	0.0	0.0	0.0			0.0		0.0	0.0	0.0
			0.0	0.0	0.0	0.0						0.0	0.0	0.0
			0.0		0.0	0.0								0.0
			0.0											0.0
														0.0
														0.0
**Acute lesions**	1.5	4.0	3.0	1.0	3.5	3.0	3.0	1.5	2.0		2.0	1.0	3.0	1.0
	2.0	1.0	1.0	1.0	1.0	3.0	2.0	1.5	3.0		1.5	1.0	3.0	3.0
	4.0	2.0	4.0	1.0	0.5	1.0	4.0	3.0	4.0		0.5	1.0	2.0	3.0
	4.0	1.0	0.5	1.0	3.0	1.0	2.5		2.0		4.0			
			2.0	4.0	0.5	4.0	0.5		2.0		3.0			
			1.0	1.0			3.0		3.0		3.0			
				3.0					4.0		2.0			
									2.0					
									2.0					
									3.5					
**Subacute lsions**	3.0	1.0	1.5	2.0	1.0	3.0	2.5	1.0	3.0	2.0	1.5	1.5		
	1.0	1.0	0.5	2.0	1.5		1.5	1.5	2.0	1.0	2.5	1.0		
	1.0	3.0	0.5		2.0		1.0	2.5	1.0	1.0	2.0	0.5		
	3.0	3.0	1.0		2.0		1.0	1.5			1.0	1.0		
		3.0	2.5					4.0			2.0	0.5		
		1.0										1.0		
		3.0												
		2.5												
		1.0												
**Chronic lesions**	1.0			1.0	3.0	3.0	2.0	2.5	3.5	0.5	4.0	2.5		
	1.5			2.0	2.0	2.0	2.0	2.0	2.0	1.5	1.5	2.0		
	3.0				2.0	1.0	3.0	1.5		2.5	2.0	1.5		
	2.0					1.0	3.0	3.0		1.5				
	2.0					1.0		1.5		2.5				
								3.0		1.5				
								3.0		0.5				
										1.0				
										1.0				
										1.5				
										1.0				
**Med**	2.0	2.0	0.5	1.0	1.0	1.0	2.0	1.5	2.0	1.0	2.0	1.0	2.0	0.0

Each number indicates the lesion score for an individual bird, and the last row shows the median of the grades in that entire column.

Table S2. Kidney lesion grades for each bird from both the infected and the in-contact groups, with each strain used in the study and at each timepoint after infection/mock infection.

	**N1/62**		**Q1/73**		**V5/90**		**N1/03**		**N1/08**		**Q1/13**		**Control**	
	**5 DPI**	**9 DPI**	**5 DPI**	**9 DPI**	**5 DPI**	**9 DPI**	**5 DPI**	**9 DPI**	**5 DPI**	**9 DPI**	**5 DPI**	**9 DPI**	**5 DPI**	**9 DPI**
	0.0	4.0	0.0	1.0	0.0	0.0	0.0	1.0	0.0	0.0	0.0	0.0	0.0	0.0
	0.0	4.0	0.0	0.0	0.0	0.0	0.0	0.0	0.0	0.0	0.0	0.0	1.0	0.0
	1.0	3.0	1.0	0.0	0.0	0.0	0.0	1.0	0.0	0.0	0.0	0.0	0.0	0.0
	0.0	4.0	0.0	0.0	0.0	0.0	0.0	0.0	2.0	0.0	0.0	0.0	0.0	0.0
	1.0	3.0	0.0	0.0	0.0	0.0	0.0	0.0	0.0	0.0	0.0	0.0	0.0	0.0
	3.0	3.0	0.0	0.0	0.0	2.0	0.0	0.0	0.0	0.0	0.0	0.0		0.0
	0.0	2.0	0.0	0.0	0.0	0.0	1.0	0.0	0.0	0.0	0.0	0.0		0.0
	3.0	3.0	0.0	0.0	0.0	0.0	0.0	0.0	0.0	0.0	0.0	0.0		1.0
	0.0	2.0	0.0	0.0	0.0	2.0	0.0	0.0	0.0	0.0	0.0	0.0		0.0
	1.0	4.0	0.0	0.0	0.0	0.0	1.0	0.0	0.0	0.0	0.0	0.0		0.0
	0.0	2.0	0.0	0.0	0.0	0.0	0.0	1.0	0.0	0.0	0.0	0.0		
	4.0	2.0	0.0	0.0	0.0	0.0	1.0	0.0	0.0	0.0	0.0	2.0		
	0.0	2.0	0.0	0.0	0.0	0.0	0.0	0.0	0.0	0.0	0.0	0.0		
	3.0		0.0		0.0	2.0	0.0	0.0	1.0	0.0	0.0	0.0		
	0.0				0.0	0.0	0.0	0.0		0.0	0.0	0.0		
**Med**	2.0	2.0	0.5	1.0	1.0	1.0	2.0	1.5	2.0	1.0	2.0	1.0	2.0	0.0

Each number indicates a score for a different bird score, and the last row shows the median of the grades in that column.

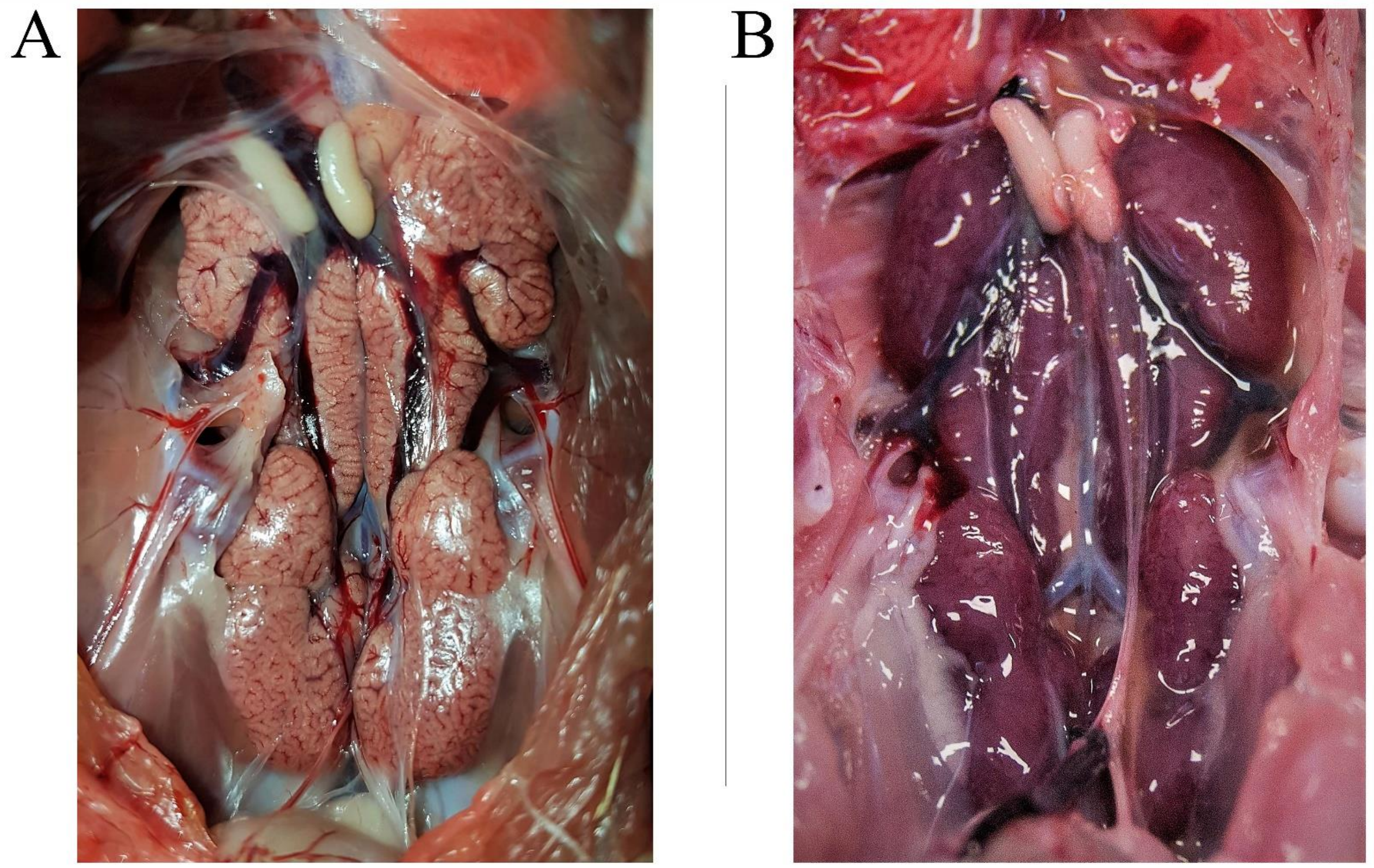

Figure S1. A, kidneys from a chicken that died in the group exposed to the strain N1/62 (T strain) of IBV. It can be noted the urate deposits, which gives the kidney a “brain” appearance. B, kidneys from a chicken that belonged to the uninfected control group, not exposed to IBV. Photos credit: Jose Quinteros
